# A Low Dose Combination of Withaferin A and Caffeic Acid Phenethyl Ester Possesses Anti-Metastatic Potential In Vitro: Molecular Targets and Mechanisms

**DOI:** 10.3390/cancers14030787

**Published:** 2022-02-03

**Authors:** Anissa Nofita Sari, Jaspreet Kaur Dhanjal, Ahmed Elwakeel, Vipul Kumar, Hazna Noor Meidinna, Huayue Zhang, Yoshiyuki Ishida, Keiji Terao, Durai Sundar, Sunil C. Kaul, Renu Wadhwa

**Affiliations:** 1 AIST-INDIA DAILAB, National Institute of Advanced Industrial Science & Technology (AIST), Central 5-41, Tsukuba 305-8565, Japan; sari-anissa@aist.go.jp (A.N.S.); elwakeela@uni.conventry.ac.uk (A.E.); hazna.meidinna@aist.go.jp (H.N.M.); zhang-huayue@aist.go.jp (H.Z.); 2 School of Integrative & Global Majors (SIGMA), Tsukuba Life Science Innovation, University of Tsukuba, Tsukuba 305-8577, Japan; 3 Indraprastha Institute of Information Technology Delhi, Okhla Industrial Estate, Phase III, New Delhi 110-020, India; jaspreet@iiitd.ac.in; 4 DAILAB, Department of Biochemical Engineering & Biotechnology, Indian Institute of Technology (IIT)-Delhi, Hauz Khas, New Delhi 110-016, India; vipul.kumar@dbeb.iitd.ac.in (V.K.); sundar@dbeb.iitd.ac.in (D.S.); 5 CycloChem Co., Ltd., 7-4-5 Minatojima-Minamimachi, Chuo-ku, Kobe 650-0047, Japan; yoshiyuki.ishida@cyclochem.com (Y.I.); keiji.terao@cyclochem.com (K.T.)

**Keywords:** ashwagandha, withaferin A (Wi-A), propolis, caffeic acid phenethyl ester (CAPE), combination Wi-ACAPE, inhibition, metastasis, angiogenesis, cancer therapy

## Abstract

**Simple Summary:**

Cancer therapy suffers from its high cost and high rate of adverse effects and relapse of the disease. Hence, the new (preferably natural), economic and safer therapeutic as well preventive measures have been on demand and have been subject of priority research. We have, earlier, demonstrated anticancer activity in the extracts of Ashwagandha leaves and propolis. A combination of Wi-A (an active anticancer ingredient in Ashwagandha extract) and CAPE (an active anticancer ingredient in propolis) was earlier shown to offer higher and cancer cell-selective cytotoxicity. In the present study, we report an anti-metastasis activity in the low dose combination of Wi-A and CAPE along with its mechanism of action and propose its use in cancer metastasis treatment.

**Abstract:**

Withaferin A (Wi-A) and Caffeic Acid Phenethyl Ester (CAPE) are the bioactive ingredients of Ashwagandha (*Withania somnifera*) and propolis, respectively. Both of these natural compounds have been shown to possess anticancer activity. In the present study, we recruited a low dose of each of these compounds and developed a combination that exhibited remarkably potent anti-migratory and anti-angiogenic activities. Extensive molecular analyses including a cDNA array and expression analyses of the specific gene targets demonstrated that such activities are mediated through their effect on cell adhesion/tight junction proteins (Claudins, E-cadherin), inhibition of canonical Wnt/β-catenin signaling pathways and the consequent downregulation of EMT-signaling proteins (Vimentin, MMPs, VEGF and VEGFR) that play a critical role in cancer metastasis. The data supported that this novel combination of Wi-A and CAPE (Wi-ACAPE, containing 0.5 µM of Wi-A and 10 µM of CAPE) may be recruited for the treatment of metastatic and aggressive cancers and, hence, warrant further evaluation by recruiting a variety of experimental and clinical metastatic models.

## 1. Introduction

Cancer is a highly heterogenous disease involving multiple mechanisms which are responsible for its proliferation, migration and stem cell characteristics. In spite of the tremendous progress that has been made in cancer research and therapy, it is coined as an uncurable disease due to its three main characteristics (i) metastasis, the phenomenon by which cancer cells move from the primary site to secondary sites in the body, (ii) drug resistance and (iii) cancer cell stemness [1,2,3]. Each of these three involves the interplay of multiple proteins in numerous ways, as well as their functional networking that influences the cancer characteristics and, hence, requires targeted treatment. Some of the major proteins and signaling pathways that contribute to the progression and metastasis of cancer include anomalous p53, PI3K/AKT/mTOR, EGFR, VEGF and Wnt/β-catenin signaling [4,5,6,7,8,9]. Furthermore, various cancer phenotypes have been shown to be regulated by the tissue microenvironment, microRNAs and epigenetic mechanisms [10,11,12].

The epithelial to mesenchymal transition (EMT) is an essential step in cancer metastasis. It allows cancer cells to acquire migratory and invasive properties, regulated by several EMT-inducing transcription factors (Twist, Snail, Slug and Zeb) that are involved in protein signaling cascades, such as p53, Akt, STAT3, MAPK, Wnt and β-catenin. EMT is largely manifested by the downregulation of E-cadherin and the upregulation of various metastatic proteins, including N-cadherin, vimentin, mortalin, matrix metalloproteinases (MMPs) and CARF [13,14,15,16,17,18]. E-cadherin is a key regulator of intercellular adhesion. It forms an essential component of the adherens junction, where it binds to β-catenin and sequesters it in the cell membrane as E-cadherin: β-catenin complex, thereby preventing β-catenin-mediated EMT signaling [19,20]. The loss of E-cadherin, in several kinds of cancers, has been shown to enhance the nuclear translocation of β-catenin, leading to an activation of Wnt/β-catenin signaling that promotes cell migration and invasiveness [21,22,23,24]. The tight junction and adherens junction consist of the transmembrane proteins (Occludin and Claudin) that regulate the movement of ions and solutes between the adhering cells and prevent the mixing of membrane proteins between the apical and basolateral membranes. Activated EMT is often characterized by (i) the loss of tight junctions and switching of E- to N-cadherin—the latter defines the mesenchymal cell characteristics [17,25,26]—and (ii) the active Wnt/β-catenin signaling pathway, a key player of metastasis signaling. β-catenin has dual functions; the regulation of intercellular adhesion and transcriptional activation of the canonical Wnt signaling pathway. An imbalance in the structural and signaling properties of β-catenin has been confirmed in several kinds of cancers [27,28,29,30]. However, the understanding of the underlying molecular mechanisms remains incomplete. The stabilization and accumulation of β-catenin in the cytoplasm and nucleus are important hallmarks of activation of Wnt signaling. In the presence of a Wnt ligand, the accumulated β-catenin translocates into the nucleus and establishes a complex with T-cell factor (TCF), a transcription factor, leading to the activation of its downstream target genes and resulting in uncontrolled cell proliferation and carcinogenesis. Cancer metastasis is influenced by matrix metalloproteinases that function in the extracellular environment, causing degradation of the matrix and non-matrix proteins. Enriched in most cancers, they are regulated by a large array of oncogenic factors [17]. Similarly, VEGF, which plays a critical role in the formation of blood and lymph vessels as well as tumor angiogenesis, is found to be upregulated in most cancers and regulated by proteins involved in hypoxia signaling and the tumor microenvironment [31,32,33]. Given these premises, Wnt/β-catenin, MMP and VEGF signaling have been suggested as cancer therapeutic targets [9,17,34].

Tumor eradication through radiation therapy, surgical resection and/or chemotherapy are the conventional regimes of cancer treatment and are established to ease the burden of cancer. However, due to the high cost of the treatment, a multitude of adverse effects and drug resistance research, the development of natural compounds for cancer treatment has been initiated. We had, earlier, reported anticancer activity in propolis and in the leaf extract of Ashwagandha (*Withania somnifera*). The active anticancer components were defined as Caffeic Acid phenethyl ester (CAPE) and Withaferin A (Wi-A), respectively. Both of these have been shown to possess multimodal anticancer activities [35]. Furthermore, we developed a combination of Wi-A and CAPE which demonstrated better anticancer potential, in human ovarian and cervical cancer cells, as compared to each of the compounds alone [36]. The combination was shown to activate p53 and inactivate PARP1 (poly ADP-ribose polymerase1), yielding the growth arrest or apoptosis of cancer cells. Several studies have reported anti-metastasis activity of Wi-A and CAPE in different models of cancer including breast, gastrointestinal, melanoma, non-small cell lung cancer and ovarian carcinoma [37,38,39,40,41,42,43]. In view of these reports, we investigated if a combination of Wi-A and CAPE at a low dose could offer anti-metastasis activity. Using human cervical cancer (HeLa) and breast cancer (MCF-7, Mortalin-OE (Mot-OE) MCF-7 and MDA-MB-231) cells as models, we investigated the anti-migration, anti-invasion and anti-angiogenesis activities of the compounds either individually or in combination. We demonstrate that a combination of Wi-A and CAPE (called Wi-ACAPE), at a low dose, possesses anti-metastatic activity and its molecular mechanism of action. Based on its anti-EMT, anti-MMP and anti-VEGF activities, Wi-ACAPE is suggested as a natural, economical and safer mixer for the inhibition and treatment of cancer metastasis.

## 2. Materials and Methods

### 2.1. Cell Culture and Drug Treatment

Human cervical cancer cells (HeLa), breast cancer cells (MCF-7, Mortalin overexpressing MCF-7 (Mot-OE MCF-7) and MDA-MB-231), normal lung fibroblasts (MRC5 and TIG-3) and umbilical vein endothelial cells (HUVEC), obtained from the Japanese Collection of Research Bioresources Cell Bank (JCRB Cell Bank, Tokyo, Japan), were cultured in Dulbecco’s Modified Eagle’s Medium (DMEM) (Invitrogen, Carlsbad, CA, USA), supplemented with 5–10% fetal bovine serum (Fujifilm WAKO Pure Chemical Corporation, Osaka, Japan) and 1% penicillin-streptomycin at 37 °C in an atmosphere of 5% CO_2_. The 5 mM-stock solutions in Dimethyl Sulfoxide (DMSO) (Fujifilm WAKO Pure Chemical Corporation, Osaka, Japan) of Wi-A and CAPE were prepared and stored in −20 °C. The compounds were diluted in cell culture media to the working concentrations of 0.5 μM (Wi-A), 10 μM (CAPE) or their combination, Wi-ACAPE (0.5 μM + 10 μM). Cells were treated with the compounds at ~70% confluency for 48 h.

### 2.2. Cell Viability Assay

**Figure 1:**
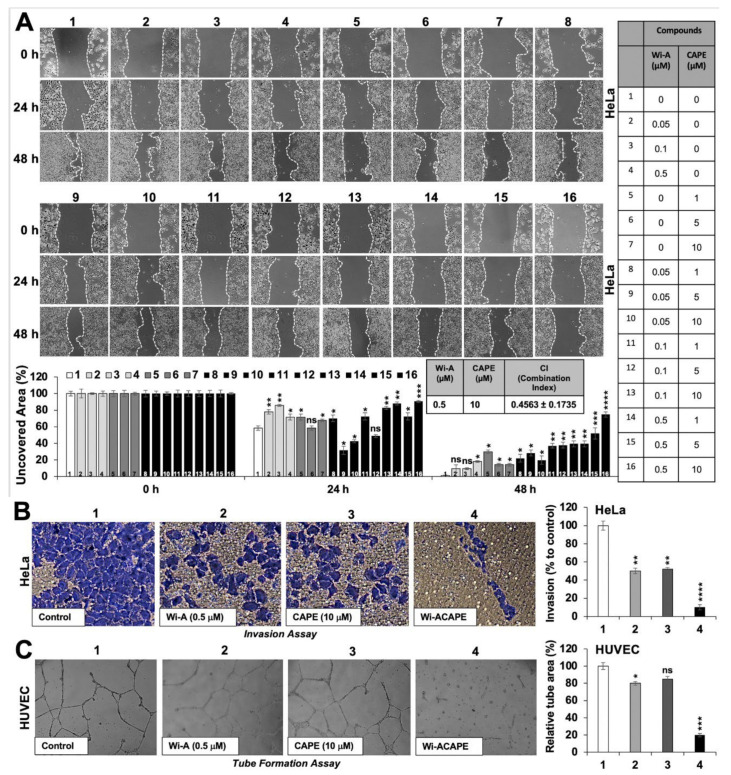
Wi-ACAPE possesses anti-metastatic potential. Treatment with a combination of Withaferin A (Wi-A) and Caffeic acid phenethyl ester (CAPE) (Wi-ACAPE) for 48 h inhibited cell migration, invasion and angiogenesis in vitro. Stronger effects on the delay of HeLa cells migration (**A**), invasion (**B**) and HUVEC cells tube formation (**C**) were observed in response to the treatment (48 h) with Wi-ACAPE than with either Wi-A or CAPE alone. Data were normalized against control and plotted as percentage difference. Each data set represented the mean SD of at least three independent experiments. Statistical significance was defined as values of *p* > 0.05 (ns), *p* ≤ 0.05 (*), *p* ≤ 0.01 (**), *p* ≤ 0.001 (***) and *p* ≤ 0.0001 (****), which represent non-significant, significant, very significant, highly significant and extremely significant, respectively.

The cytotoxicity of Wi-A, CAPE and their combinations were determined by quantitative cell viability assay using MTT [3-(4,5-dimethylthiazol-2-yl)-2,5-diphenyltetrazolium bromide] assay (Sigma Aldrich, Tokyo, Japan). Cells were seeded in 96-well plates (TPP®, Trasadingen, Switzerland) at a density of 5 × 10^3^ cells/well and incubated for 24 h at 37 °C in a CO_2_ incubator. The cells were treated with Wi-A, CAPE or their combinations (as described in Figure S1 and Figure 1) for 48 h, followed by the addition of MTT solution (0.5 mg/mL; 100 μL/well) incubation at 37 °C for a further 3–4 h and then the addition of 100 μL DMSO. The plates were shaken for 10 min for the proper dissolution of the formed formazan crystals. The absorbance was measured at 570 nm using an Infinite M200 Pro microplate reader (Tecan Group Ltd., Männedorf, Switzerland).

### 2.3. Morphological Observations

Cells (15 × 10^4^/well) were seeded in the 6-well plates (TPP, Trasadingen, Switzerland) and allowed to settle overnight followed by incubation with the compounds for 48 h. Cell morphology was observed, and the images were captured using a Nikon TS100-F phase contrast microscope (Nikon, Tokyo, Japan).

### 2.4. In Vitro Scratch/Wound Healing Assay

HeLa, MCF-7, Mot-OE MCF-7 and MDA-MB-231 cells (1 × 10^5^ cells/well) were plated in 6-well plates (TPP, Trasadingen, Switzerland) and allowed to form monolayers through the overnight incubation at 37 °C in a CO_2_ incubator. A linear wound was created by manually scraping the cell monolayer with a p200 pipette tip. The cells were washed three times with PBS and were cultured either in the control culture medium or test compounds-supplemented medium (Wi-A, CAPE and their combinations as shown in Figure 1A). The movement of cells into the scratched area were observed and captured using a Nikon TS100-F microscope (Nikon, Tokyo, Japan) at 0, 24 and 48 h.

### 2.5. In Vitro Cell Invasion Assay

The in vitro cell invasion assay was carried out using a Corning BioCoat Matrigel Invasion Chamber kit (354480; Corning, Labware, Inc., Two Oak Park, MA, USA). HeLa cells suspended in 0.5 mL serum-free Dulbecco’s modified Eagle’s medium (DMEM) were plated into the top of the invasion inserts. The 0.75 mL of 10% fetal bovine serum-supplemented DMEM was added into the well of the 24-well plate as a chemoattractant. After 48 h of incubation, the inserts were transferred to new plates and washed with PBS. Fixation of the cells suspended in the Matrigel basement membrane matrix at the bottom of each insert was performed using methanol:acetone (1:1). Then, cells were stained overnight with 0.5% Crystal Violet. The excessive stain was removed by rinsing in distilled water. The inserts were air-dried, visualized and photographed under the microscope (Nikon TS100-F, Nikon, Tokyo, Japan). DMSO was used as a vehicle control.

### 2.6. Tube Formation Assay

The anti-angiogenic activity of Wi-A, CAPE and their combination, Wi-ACAPE, was assessed through the formation of Human Umbilical Vein Endothelial cells (HUVECs) tube-like structures on a basement membrane matrix. The 24-well (16 mm diameter) tissue culture plates were coated with 250 μL of the BD Matrigel growth factor-reduced solution (BD Biosciences, Franklin Lakes, NJ, USA) and incubated at 37 °C for 30 min until the Matrigel became solid. HUVECs were seeded in the Matrigel-coated plates at a density of 1.5 × 10^5^ cells/well and were cultured either in M199 medium (control) or test compounds-supplemented M199 medium. Images of the tubular structures were captured; the area covered by the tube networks was measured by Image-Pro Plus 6.0 software (Media Cybernetics, Silver Spring, MD, USA).

### 2.7. cDNA Array

Total RNA was isolated from control and treated HeLa cells using RNeasy mini kit (Qiagen, Stanford Valencia, CA, USA) following the manufacturer’s protocol. The quantity and quality of RNA was measured by using NanoDropTM (Nanodrop ND-1000 Spectrophotometer) (NanoDrop Technologies, Inc., Wilmington, DE, USA) and Agarose gel electrophoresis, respectively. Equal amounts of total RNA samples (1 µg) were labelled with Cy3 or Cy5 and subjected to cDNA microarray (Cell Innovator Inc., Fukuoka, Japan). The cDNA was amplified, labeled using a Low Input Quick Amp Labeling Kit (Agilent, #5190-2305, Santa Clara, CA, USA) and hybridized to a 60K Agilent 60-mer oligomicroarray (SurePrint G3 Human Gene Expression Microarray 8 × 60K v3) according to the manufacturer’s instructions. All hybridized microarray slides were scanned by an Agilent scanner. Relative hybridization intensities and background hybridization values were calculated using Agilent Feature Extraction Software (v9.5.1.1).

The signal values obtained for each sample were used to compute the change in expression of different genes after treatment with respect to the control group. Firstly, log2fold change values were computed for Wi-A, CAPE and Wi-ACAPE combination treated cells with respect to the untreated control cells. The genes with a log2fold change greater than 1 or less than −1 were considered up-and down-regulated, respectively. The enrichment of these dysregulated genes in different pathways was then carried out using The Database for Annotation, Visualization, and Integrated Discovery (DAVID) v6.8 (https://david.ncifcrf.gov/ (accessed on 18 August 2021)).

### 2.8. Flow Cytometry Analysis

The cell surface expression of VEGFR1 and VEGFR2 in control and treated HeLa cells were detected using a Cell Surface Staining Flow Assay Kit (Novus Biologicals, LLC, Englewood, CO, USA) following the manufacturer’s instructions. The expression analysis was performed using Guava PCA flow cytometer (Millipore, Billerica, MA, USA) following the manufacturer’s protocol.

### 2.9. Western Blot Analysis

Control and treated cells were harvested after 48 h, lysed using RIPA Lysis Buffer (Thermo Fisher Scientific, Waltham, MA, USA) supplemented with cOmplete^TM^, Mini protease inhibitor cocktail (Roche Applied Science, Mannheim, Germany). Then, total cell lysates were vortexed in a cold room (4 °C) for 30 min. Lysates were centrifuged at 15,000 rpm for 15 min. The supernatant was subjected to BCA protein assay (Thermo Fisher Scientific, Waltham, MA, USA) to determine the protein concentration of each sample. Equal amounts of total proteins (10–20 µg) were separated in 6–12% SDS-polyacrylamide gel electrophoresis (SDS-PAGE), then transferred to a polyvinylidene difluoride (PVDF) membrane (Millipore, Billerica, MA, USA) using a semi dry transfer blotter (ATTO Corporation, Tokyo, Japan) or a wet transfer using Mini-PROTEAN Tetra Cell (BIO-RAD, Hercules, CA, USA). The membrane was blocked using 3% of Bovine Serum Albumin Fraction V (Fujifilm WAKO Pure Chemical Corporation, Osaka, Japan) at room temperature for 60–120 min. Blocked membranes were probed with the target protein-specific primary antibodies overnight at 4 °C. Primary antibodies were: E-cadherin (24E10), β-catenin (D13A1), phospo-p38 MAPK (D3F9), Phospo-Akt (D9E), AKT (C67E7), Phospho-c-Raf (56A6), p44/42 MAPK (Erk ½) (137F5), FAK (D5O7U), PI3 Kinase p110 α (C73F8), hnRNP-K (R332) and MMP9 (G657) (Cell Signaling Technology, Danvers, MA, USA); Claudin 1(A-9), Wnt-1 (E-10), Cyclin D1 (DSC-6), N-cadherin (D4R1H), VEGFR2 (A-3), MMP1 (3B6), MMP2 (2C1), MMP 3/10 (F-10), MMP7 (FL-267), Fibronectin (568) and Vimentin (V-9), (Santa Cruz Biotechnology, Paso Robles, CA, USA); c-Myc (ab32072), VEGFA (ab46154), VEGFR1 (ab32152) (Abcam, Cambridge, UK); Mortalin (37-6) [44] and CARF (A-10) [45] antibodies were generated in our laboratory. After three washes in TBS-T, the blots were incubated with horseradish peroxidase (HRP)-conjugated secondary antibodies (anti-rabbit IgG and anti-mouse IgG (Santa Cruz Biotechnology, CA, USA) and developed using the enhanced chemiluminescence system (GE Healthcare, Buckinghamshire, UK). Anti-β-actin antibody (Abcam, Cambridge, UK) was used as an internal loading control. The protein band images were analyzed by ImageJ 1.46 software (National Institutes of Health, Bethesda, MD, USA).

### 2.10. Immunofluorescence

HeLa cells (4 × 10^4^ cells/well) were plated on 18-mm glass coverslips placed in 12-well plates and allowed to settle overnight. After 24 h, the cells were treated with Wi-A, CAPE or their combination for 48 h, then washed twice with PBS and fixed in methanol: acetone (1:1) solution at 4 °C for 5–10 min. Cells were then washed with PBS. Permeabilization of the cells was performed using PBS with 0.1% Triton X-100 (PBS-T) for 10 min, followed by blocking using 2% of Bovine Serum Albumin Fraction V in PBS-T at room temperature for 1 h. Fixed cells were incubated with primary antibodies (as listed above in Western blotting analysis section). Immunostaining was visualized through staining (incubation for 1–2 h) with secondary antibodies conjugated with either Texas RED (Amersham Biosciences, Buckinghamshire, UK) or fluorescein isothiocyanate (FITC), Alexa-488, and Alexa-594 (Molecular Probes, Eugene, OR, USA). Hoechst 33342 (Invitrogen, Molecular Probes, Eugene, OR, USA) was used for counter staining for the nucleus. The coverslips were mounted on glass slides and examined under a Zeiss Axiovert 200 M microscope with AxioVision 4.6 software (Carl Zeiss, Tokyo, Japan) and confocal laser scanning microscope (LSM510, Carl Zeiss, Tokyo, Japan). Protein expression represented by the fluorescence signals was quantified using ImageJ 1.46 software (National Institutes of Health, Bethesda, MD, USA).

### 2.11. Immunoprecipitation

Control, Wi-A, CAPE and Wi-ACAPE treated cells were harvested and lysed using the non-ionic NP-40 buffer. The protein concentrations of whole-cell lysates were determined using BCA assay (Thermo Fisher Scientific, Rockford, IL, USA). Cell lysates containing 300–500 μg of total protein were incubated (4 °C overnight in slow rotation) with either control IgG (sc-2025) (Cell Signaling Technology, Danvers, MA, USA) or anti-E-cadherin antibody (67A4) (Cell Signaling Technology, Danvers, MA, USA) and VEGFA (ab46154) (Abcam, Cambridge, UK) overnight followed by the addition of A/G PLUS-Agarose beads (sc-2003) (Santa Cruz Biotechnology, Paso Robles, CA, USA). The mixture was incubated at 4 °C for 4 h in slow rotation and was then centrifuged at 2500 rpm at 4 °C for 5 min to collect immunoprecipitant. The supernatants were removed. Pellets containing beads and immunoprecipitant were washed thrice with NP-40 buffer. Then, pellets were mixed with SDS buffer and boiled at 96 °C for 10 min. Immunoprecipitants were resolved on SDS-PAGE and then transferred to a PVDF membrane. The protein of interest was detected as described previously in the Western blotting’s section.

### 2.12. VEGF ELISA

VEGFA antibody (ab46154) (Abcam, Cambridge, UK) was diluted to a final concentration of 4–8 μg/mL in ELISA Coating buffer (421701) (BioLegend Inc., BioLegend Way, San Diego, CA, USA) and incubated overnight at 4 °C in ELISA plates (Corning, Labware, Inc., Two Oak Park, MA, USA). The wells were then washed thrice with PBS containing 200 µL 0.05% Tween 20 (PBS-T) and blocked with 5% of bovine serum albumin at room temperature for 1 h. HeLa cells (15 × 10^4^/well) were seeded in 6-well plates and allowed to settle overnight, followed by treatment with Wi-A, CAPE or Wi-ACAPE. Cells were washed with PBS, collected by trypsinization and centrifuged at 1500 rpm (at 4 °C) for 10 min. Extraction buffer (100 mM Tris, pH 7.4, 150 mM NaCl, 1 mM EGTA, 1 mM EDTA, 1%, Triton X-100, 0.5% Sodium deoxycholate) containing cOmplete^TM^, Mini protease inhibitor cocktail (Roche, Basel, Switzerland) was used for cell lysis (incubation at 4 °C for 1 h). The cell lysates (75–100 µg in 100 µL) and the standard were incubated at room temperature for 4 h in a 96-well VEGFA coated ELISA plate. Wells were then washed with PBS-T thrice, reloaded with an anti-VEGFA antibody (100 ng/mL) in 100 µL diluent buffer (1.7 mM Na_2_CO_3_, 3.3 mM NaHCO_3_ in distilled water pH 9.6) at room temperature for 1 h, followed by three PBS-T washing and, finally, they were incubated with the secondary anti-rabbit horseradish peroxidase (HRP)-conjugated antibody (Thermo Fisher Scientific, Waltham, MA, USA) at room temperature for 30 min. The plate was washed thrice with PBS-T incubated with 100 µL 3,3′,5,5′-tetramethylbenzidine (TMB) substrate (421101) (BioLegend Inc., BioLegend Way, San Diego, CA, USA) for 30 min, followed by the addition of 100 µL Stop Solution (423001) (BioLegend Inc., BioLegend Way, San Diego, CA, USA). Optical density was measured at 450 nm using an Infinite M200 Pro microplate reader (Tecan Group Ltd., Mannedorf, Switzerland). Differences in VEGFA absorbance at a wavelength of 450 nm were then expressed and plotted in percentage, taking control as 100% and using Microsoft™ Office.

### 2.13. Apoptosis Assay

HeLa cells were seeded in 6-well plates. After 48 h, control and Wi-A/CAPE/Wi-ACAPE-treated cells were collected by centrifugation at 3000 rpm at 4 °C for 5 min. The cell pellets were resuspended with 100 μL fresh media and stained with Guava Nexin Reagent (EMD Millipore Corporation, Berlington, MA, USA). Apoptosis analyses were done by the Guava PCA-96 System (Luminex Corporation, Austin, TX, USA). Apoptotic cells were detected and quantified by FlowJo software (v7.6, Flow Jo, LLC, Ashland, OR, USA).

### 2.14. Combination Index (CI) Analysis

The Chou–Talalay’s combination index (CI) method using CompuSyn software, Paramus, NJ (2005) [46] was performed to analyze the optimal combination ratio of Wi-A and CAPE. The CI score represents quantitative determination of the synergism (CI < 1), antagonism (CI > 1) and additive effect (CI = 1) of drug combination.

### 2.15. RNA Extraction and Real Time Quantitative Polymerase Chain Reaction (RT-qPCR)

Total RNA was extracted from control and treated HeLa cells using an RNeasy mini kit (Qiagen, Stanford Valencia, CA, USA) following the manufacturer-described protocol. Equal amounts of total RNA (1 µg) from samples were reverse transcribed into cDNA using QuantiTect Reverse Transcription kit (Qiagen, Tokyo, Japan) following the manufacturer’s described protocol. Gene expression quantification was performed by real time quantitative PCR (RT-qPCR) using SYBR Select Master mix (Applied Biosystem, Life Technologies, Foster City, CA, USA). The condition of RT-qPCR, using gene specific primers (Table S1), was 50 °C for 2 min, then 95 °C for 10 min followed by 40 cycles of denaturation (95 °C, 15 s), annealing (60 °C, 1 min) and extension (72 °C, 15 s). The relative expression level of target genes was normalized against an 18S gene as an internal control. Details of the primers used for RT-qPCR assays are provided in Table S1.

### 2.16. Statistical Analysis

Statistical data from three or more independent experiments were expressed as mean ± standard deviation. An unpaired Student *t*-test (GraphPad Prism, GraphPad software, San Diego, CA, USA) was performed to determine statistical significance between the control and experimental samples. Values of *p* > 0.05 (ns), *p* ≤ 0.05 (*), *p* ≤ 0.01 (**), *p* ≤ 0.001 (***) and *p* ≤ 0.0001 (****) were considered non-significant, statistically significant, very significant, highly significant and extremely significant, respectively.

## 3. Results

### 3.1. A Low Dose Combination of Wi-A and CAPE Inhibited Cancer Cell Migration, Invasion and Angiogenesis

We had previously reported that the combinatorial dose of Wi-A and CAPE (1 μM and 20 μM, respectively) caused selective toxicity to cancer cells through the activation of DNA damage and apoptosis signaling [36]. The effect of the combination was remarkably better than the individual compounds. The combination of Wi-A and CAPE (1 μM and 20 μM, respectively) showed a stronger effect on human cervical cancer (HeLa) cells as compared to other cancer cell lines [36]. Since HeLa cells have been widely used as a model metastatic cervical cancer cell line [47,48,49,50], we continued to use HeLa cells in the present study. The effect on HeLa was also compared with highly metastatic breast cancer cell lines (MCF-7, Mot-OE MCF-7 and MDA-MB-231) [14,51,52,53,54]. To examine the anti-metastatic activity of the combination, we recruited low doses of each of these compounds that did not cause cytotoxicity and investigated their effect on cancer cell migration, invasion and angiogenesis (the three critical phenotypes for cancer metastasis). The low non-toxic doses were determined by determining the dose-dependent cytotoxicity of Wi-A (0.05, 0.1 and 0.5 μM), CAPE (1, 5 and 10 μM) and their combinations in HeLa, MDA-MB-231, MCF-7 and Mot-OE MCF-7 cells by MTT assay. Human normal fibroblasts (MRC5 and TIG-3) were also used for comparison. As shown in Figure S1A,B, the selected doses of the compounds either alone or in combination did not show cytotoxicity to any of the cell lines examined. Direct observations of treated cells under the microscope did not reveal any stress phenotype such as detachment, condensation or blebbing, etc. We therefore selected a combination of Wi-A (0.5 μM) and CAPE (10 μM) and determined its effect on apoptosis and growth arrest by flow cytometric and molecular analyses. As shown in Figure S2A, no effect on cell growth or apoptosis was observed. Furthermore, there was no change in the expression of the proteins (p53, PARP-1 and Bcl-2) involved in growth arrest/apoptosis (Figure S2B). Based on these data, Wi-A (0.5 μM), CAPE (10 μM) and their combination were considered as being in the low non-toxic range and used for further experiments. Of note, Wi-A (0.5 μM), CAPE (10 μM) or their combination-treated normal fibroblast did not show any change in the expression of MMP3 and vimentin proteins that are involved in the metastasis (Figure S2C).

We next examined the migration capacity of control and treated cells by scratch/wound healing assay.

As shown in Figure 1A, HeLa cells treated with either Wi-A or CAPE showed slower migration into the wounded area, suggesting that each of these two compounds caused inhibition of cell migration. Of note, the cells treated with Wi-A and CAPE in combination showed significantly higher anti-migratory potential; the combination (0.5 μM Wi-A + 10 μM CAPE; called Wi-ACAPE hereafter) showed the highest anti-migratory effect and was hence used for further analyses. We used the Chou–Talalay analysis method to quantify the pharmacodynamic interaction of Wi-ACAPE [46,55]. The results supported that Wi-ACAPE exhibited synergistic in vitro pharmacodynamic interaction between Wi-A and CAPE and possessed a Combination Index (CI) value of 0.4563 (less than 1) (Figure 1A). These data demonstrated that Wi-ACAPE offers a synergistic anti-migratory potential. In order to rule out that the effect was not cell line specific, we also used other metastatic cell lines (MCF-7, Mot-OE MCF-7 and MDA-MB-231 cells). As shown in Figure S3A,C,E, Wi-ACAPE caused a stronger inhibition of cell migration in all three cell lines.

We next examined anti-invasion and anti-angiogenic potentials of Wi-ACAPE through Boyden Chamber and tube formation assays, respectively. HUVEC cells that have been widely recognized as an excellent in vitro model to study angiogenesis were recruited for tube formation assay [56,57,58,59]. As shown in Figure 1B, compared to the untreated control cells, cells treated with either Wi-A (0.5 μM) or CAPE (10 μM) showed inhibition in cell invasion. Wi-ACAPE treated cells showed a remarkable reduction in cell invasion. In agreement with these data, the HUVEC cell tube formation assay also showed a decrease in tube formation in either Wi-A or CAPE treated cells. Of note, Wi-ACAPE treated cells showed a complete absence of tube formation (Figure 1C). Taken together, these data demonstrated that a low dose combination of Wi-A and CAPE (Wi-ACAPE) possesses significant anti-metastasis and anti-angiogenic activities.

### 3.2. Wi-ACAPE Treated Cells Showed Inactivation of Metastatic Signaling Pathways

**Figure 2:**
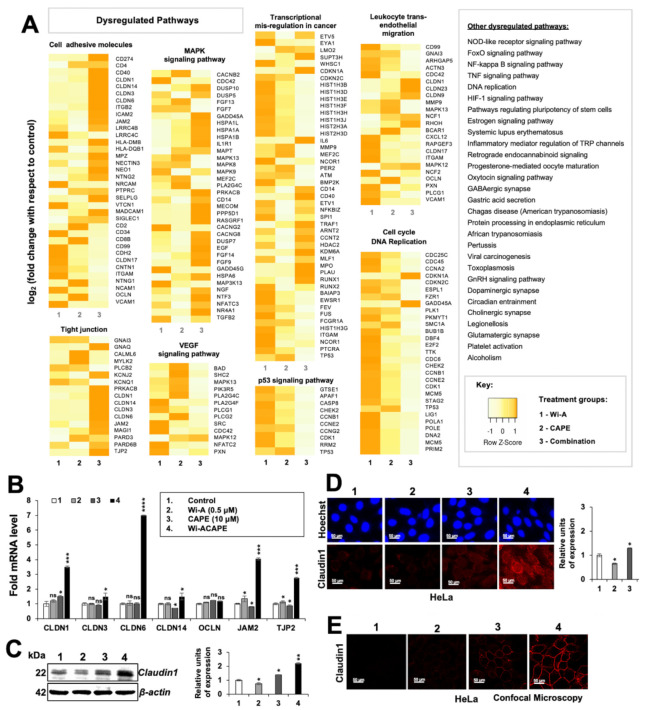
cDNA array analysis of Wi-A/CAPE/Wi-ACAPE treated cells. Wi-A/CAPE/Wi-ACAPE caused dysregulation of cell adhesion molecules (CAMs), activation of tight junction proteins and downregulation of vascular endothelial growth factor (VEGF) and others signaling pathways that are involved in the cancer metastasis, as shown in the cDNA array result (**A**). Increased mRNA expressions of the members of the claudin family, junctional adhesion molecule 2 (JAM2) and tight junction protein (TJP2) were observed in the HeLa cells treated with Wi-ACAPE for 48 h (**B**). Wi-ACAPE caused a remarkable increase in Claudin 1 expression as shown by Western blotting (**C**), immunostaining (**D**) and confocal microscopy (**E**). Data were normalized against control and plotted as fold difference. Each data set represented the mean SD of at least three independent experiments. Statistical significance was defined as values of *p* > 0.05 (ns), *p* ≤ 0.05 (*), *p* ≤ 0.01 (**), *p* ≤ 0.001 (***) and *p* ≤ 0.0001 (****), which represent non-significant, significant, very significant, highly significant and extremely significant, respectively. The uncropped blots are shown in Figure S5.

We next investigated the molecular mechanisms of anti-metastatic activity of Wi-ACAPE. Control and treated (Wi-A 0.5 μM; CAPE 10 μM and Wi-ACAPE) HeLa cells were subjected to cDNA array. Although, in general practice, a fold change of ±4 that corresponds to a log_2_(fold change) of ±2 is used a threshold to account for dysregulated genes, we selected a threshold of fold change ±2 or log_2_(fold change) of ±1 to ensure the wider analysis. The up- or down-regulated genes were then subjected to functional annotation/gene enrichment analysis using DAVID server. The functional annotation clustering algorithm of DAVID integrates the techniques of Kappa statistics to measure the degree of the common genes between two annotations, and fuzzy heuristic clustering to classify the groups of similar annotations according to Kappa values. Accordingly, the more common genes that annotations share, the higher the chances are that they will be grouped together. The *p*-values associated with each annotation term inside every cluster was computed by the software using a modified Fisher Exact test, called EASE Score. Analysis of the array data revealed dysregulation of several pathways involved in carcinogenesis, cell attachment, migration and invasion. These included genes involved in cell cycle and DNA replication, trans-endothelial migration, MAPK, VEGF, p53, NF-Kβ, TNF-α and HIF-1α signalings (Figure 2A). Of note, whereas genes involved in cell cycle and DNA replication showed downregulation, many genes involved in cell adhesion showed upregulation in Wi-ACAPE-treated cells as compared to the control-, Wi-A- or CAPE-treated cells. Many genes that regulate tight junctions and MAPK signaling also showed significant upregulation in Wi-ACAPE treated cells. On the other hand, most genes involved in trans-endothelial migration showed strong downregulation. Genes involved in VEGF and p53 signaling also showed significantly higher downregulation in Wi-ACAPE-treated cells. In considering these data, we hypothesized that Wi-ACAPE-induced inhibition of cell migration may operate through its effects on the cell adhesion-tight junctions signaling pathway and, therefore, undertook its validation by expression analyses of several genes along with others (Vascular endothelial growth factor-VEGF and its receptors) that play a critical role in angiogenesis and metastasis as described in the next sections.

### 3.3. Wi-ACAPE Dysregulated Tight Junction (TJ) Genes

Based on the cDNA array results, we next investigated the effect of Wi-A, CAPE and their combination on the expression of several tight junctions (TJ) genes that play an important role in cell-to-cell adhesion, tissue integrity and metastasis [60,61].

As shown in Figure 2B, compared to the untreated control cells, CAPE-treated and Wi-ACAPE-treated HeLa cells showed an increase in the expression of the CLDN1 gene. The expression of CLDN3 and CLDN6 genes increased only in the Wi-ACAPE-treated cells. Although Wi-A-treated HeLa cells did not show a significant change in the expression of CLDN14 gene, they showed a decrease in CAPE-treated cells and increased significantly in the Wi-ACAPE-treated cells as compared to the untreated control. Additionally, the expression of JAM2 and TJP2 genes showed a minor increase in either the Wi-A- or CAPE-treated cells; Wi-ACAPE-treated cells showed a remarkable increase. On the other hand, OCLN gene expression remained unchanged in either of the treated groups (Figure 2B). Since Claudin 1 protein is an established key component of tight junction complexes [62,63], we extended the analysis to determine its level in control and treated cells. As shown in Figure 2C and Figure S3B,D,F, Claudin 1 expression showed a significant increase in Wi-ACAPE-treated cells as compared to the untreated, Wi-A or CAPE-treated HeLa, MCF-7, Mot-OE MCF-7 and MDA-MB-231 cells. Immunocytochemistry also confirmed these findings, wherein we observed an increase in membrane localized Claudin 1 protein by confocal microscopy (Figure 2D,E). In contrast, the cells treated with a high-dose combination of Wi-A and CAPE (1 μM Wi-A + 20 μM CAPE) showed cytotoxicity. Whereas expression analyses of control and treated cells showed decrease a in E-cadherin; Claudin 1, MMP3 and Vimentin did not show any significant change (Figure S4A,B).

### 3.4. Wi-ACAPE Treated Cells Showed Upregulation of E-Cadherin and Downregulation of β-Catenin

**Figure 3:**
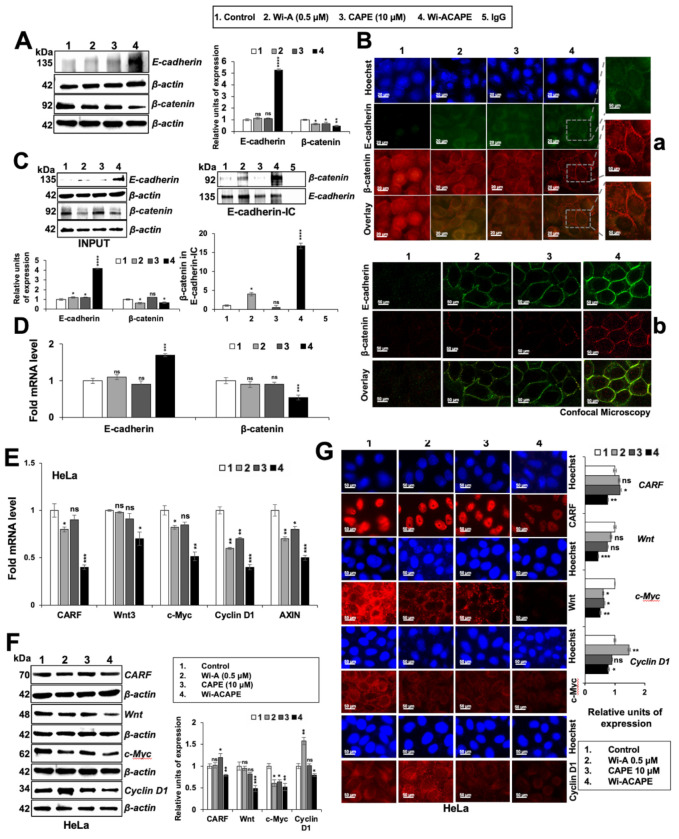
Wi-ACAPE caused reversal of EMT signaling and inactivation of Wnt/β-catenin. Wi-ACAPE modulated the expression of E-cadherin and β-catenin, leading to suppression of Wnt/β-catenin-mediated EMT signaling. Wi-ACAPE-treated (48 h) HeLa cells exhibited an increase in E-cadherin expression and a decrease in β-catenin expression as detected by Western blotting (**A**), immunocytochemistry (**B**) and confocal microscopy (**B**). Co-immunoprecipitation analyses using anti-E-cadherin-specific antibody showed an increase in the β-catenin fraction in E-cadherin complex immunoprecipitated from Wi-ACAPE-treated (48 h) HeLa cells (**C**). An increase in E-cadherin and a decrease in β-catenin mRNA levels was observed after 48 h treatment with Wi-ACAPE (**D**). Wi-ACAPE-treated (48 h) HeLa cells showed downregulation of CARF, Wnt, c-Myc, Cyclin D1 and AXIN at the mRNA (**E**) and the protein (**F**,**G**) level. Data were normalized against control and plotted as fold difference. Each data set represented the mean SD of at least three independent experiments. Statistical significance was defined as values of *p* > 0.05 (ns), *p* ≤ 0.05 (*), *p* ≤ 0.01 (**), *p* ≤ 0.001 (***) and *p* ≤ 0.0001 (****), which represent non-significant, significant, very significant, highly significant and extremely significant, respectively. The uncropped blots are shown in Figures S5 and S6.

Epithelial to Mesenchymal Transition (EMT) is an initial step in cancer metastasis. It is marked by loss of E-cadherin expression or its delocalization at cell–cell contacts [22]. In light of the above cDNA array data and validation of some genes, we next investigated E-cadherin expression (at both transcript and protein levels). As shown in Figure 3A,B, Wi-A- and CAPE-treated HeLa cells, as compared to the untreated controls, did not show a significant change in E-cadherin expression. Wi-ACAPE-treated cells, on the other hand, showed a remarkable increase. Wi-ACAPE-treated MCF-7/Mot-OE MCF-7/MDA-MB-231 cells also showed a significant increase in E-cadherin expression compared to treatment with either Wi-A or CAPE alone (Figure S3B,D,F). E-cadherin mediates intercellular adhesion through its interaction/binding with β-catenin. Under normal physiological conditions, β-catenin is sequestered to the cell membrane by E-cadherin in the E-cadherin/β-catenin complex, preventing EMT progression. The loss of E-cadherin promotes the release of β-catenin from the membrane complexes, resulting in its nuclear translocation and activated Wnt/β-catenin signaling that leads to increased cell migration and invasive characteristics of cancer cells [22,23,24,64]. Accordingly, we examined the expression and subcellular localization of β-catenin. As shown in Figure 3A and Figure S3B,D,F, both Wi-A and CAPE-treated HeLa/MCF-7/Mot-OE MCF-7/MDA-MB-231 cells showed a decrease in β-catenin expression as compared to untreated control cells, and Wi-ACAPE treated cells showed a profound decrease in β-catenin expression. We next examined the subcellular localization of E-cadherin and β-catenin in control and treated cells by immunocytochemistry. As shown in Figure 3B(a), β-catenin was seen predominantly in the nucleus in the control untreated cells. Wi-A- and CAPE-treated cells showed a decrease in nuclear β-catenin staining; Wi-ACAPE-treated cells showed predominant non-nuclear staining and its concentration in the cell membrane. We confirmed these results by confocal microscopy (Figure 3B(b)), which revealed an increase in E-cadherin in treated cells; in agreement with the Western blotting data, a remarkable increase in Wi-ACAPE treated cells was detected. E-cadherin and β-catenin colocalization was observed in the cell membrane. Furthermore, Wi-ACAPE-treated cells showed clear sequestration of β-catenin in the membrane, suggesting an increase in E-cadherin: β-catenin complex in these cells. We next investigated such complexes in control and treated cells by co-immunoprecipitation. E-cadherin complex was immunoprecipitated from control and treated cell lysates using an anti-E-cadherin specific antibody. The presence of β-catenin in E-cadherin complex was detected by probing with anti β-catenin antibody. As shown in Figure 3C, a significant increase in β-catenin in E-cadherin complex was detected in Wi-ACAPE-treated cells as compared to the untreated control, Wi-A or CAPE-treated cells. We also examined the mRNA expression of E-cadherin and β-catenin by RT-qPCR in control and treated cells. As shown in Figure 3D, Wi-ACAPE treated cells showed a ~4-fold increase in E-cadherin expression; cells treated with either Wi-A or CAPE did not show any significant change. Similarly, a decrease in β-catenin was observed in Wi-ACAPE- but not Wi-A/CAPE-treated cells. Taken together, these data suggested that Wi-ACAPE have multimodal activity that operates at the protein as well as mRNA level. An increase in E-cadherin is upregulated at the transcriptional level, causing sequestration of β-catenin in the cell membrane and inhibition of its transcriptional activation function and metastasis signaling.

### 3.5. Wi-ACAPE Caused Downregulation of Wnt/β-Catenin Mediated EMT Signaling

Considering the above data that Wi-ACAPE-mediated upregulation of E-cadherin caused the accumulation of β-catenin in the cell membrane and inhibition of transcriptional activation function, we next examined the expression of downstream effectors of β-catenin. Consistent with the upregulation of E-cadherin, its downstream negatively regulated effector and Wnt protein showed higher downregulation in Wi-ACAPE, as compared to the cells treated with either Wi-A or CAPE. Since the Wnt/β-catenin pathway is also regulated by c-Myc, cyclin D1, AXIN, CD44 and VEGF [34,65], we next examined the status of these proteins in control and treated cells. As shown in Figure 3E, Wi-A/CAPE/Wi-ACAPE-treated cells showed a decrease in Cyclin D1 and AXIN mRNA. c-Myc mRNA showed a decrease in Wi-A/Wi-ACAPE-treated cells. On the other hand, Wnt3 mRNA showed a decrease only in Wi-ACAPE (Figure 3E). Furthermore, the expression of CARF (collaborator of p14^ARF^)/CDKN2AIP mRNA that has been shown to regulate Wnt/β-catenin mediated EMT in cancer cells [18] showed a significant decrease in Wi-ACAPE-treated cells. Of note, whereas Wi-A caused downregulation of CARF, CAPE was ineffective. The protein expression analysis (Western blotting and immunostaining) revealed similar data. Wi-ACAPE-treated cells showed strong downregulation of CARF, Wnt, c-Myc and Cyclin D1 as compared to Wi-A or CAPE alone (Figure 3F). Wi-ACAPE-treated cells showed a significant decrease in c-Myc, Cyclin D1 expression as compared to control and Wi-A/CAPE-treated cells. These data were confirmed by immunocytochemistry (Figure 3G).

### 3.6. Wi-ACAPE Treated Cells Showed Reversal of EMT Signaling

**Figure 4:**
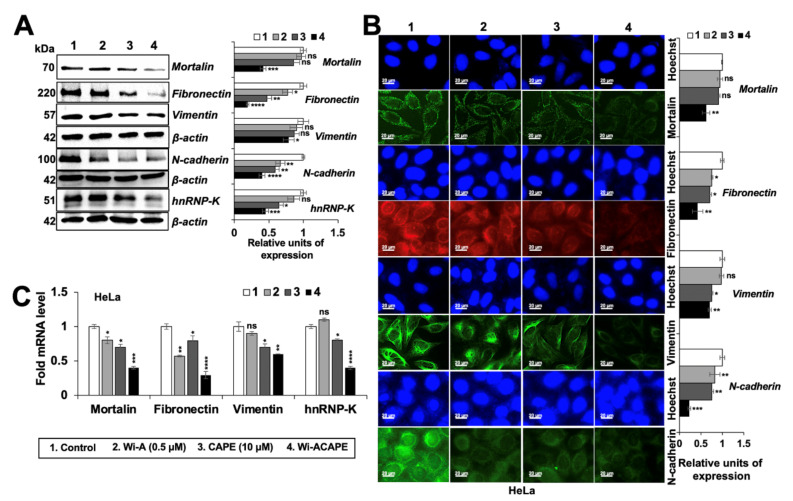
Wi-ACAPE caused reversal of EMT signaling and downregulation of mesenchymal proteins. Western blotting (**A**), immunocytochemistry (**B**) and RT-qPCR (**C**) analyses showed downregulation of Fibronectin, Vimentin, N-cadherin, hnRNP-K and Mortalin after treatment with Wi-ACAPE for 48 h. Data were normalized against control and plotted as fold difference. Each data set represented the mean SD of at least three independent experiments. Statistical significance was defined as values of *p* > 0.05 (ns), *p* ≤ 0.05 (*), *p* ≤ 0.01 (**), *p* ≤ 0.001 (***) and *p* ≤ 0.0001 (****), which represent non-significant, significant, very significant, highly significant and extremely significant, respectively. The uncropped blots are shown in Figure S7.

Based on the above results showing an increase in epithelial proteins in Wi-ACAPE-treated cells, we next examined the expression level of mesenchymal (Fibronectin, N-cadherin, Vimentin and the matrix metalloproteinase-MMPs) proteins. Consistent with the reversal of EMT signaling, the expression of the mesenchymal proteins (Fibronectin, Vimentin, N-cadherin) decreased significantly in the Wi-ACAPE-treated cells as compared to ones treated with either Wi-A or CAPE (Figure 4A, Figure S3B,D,F).

**Figure 5:**
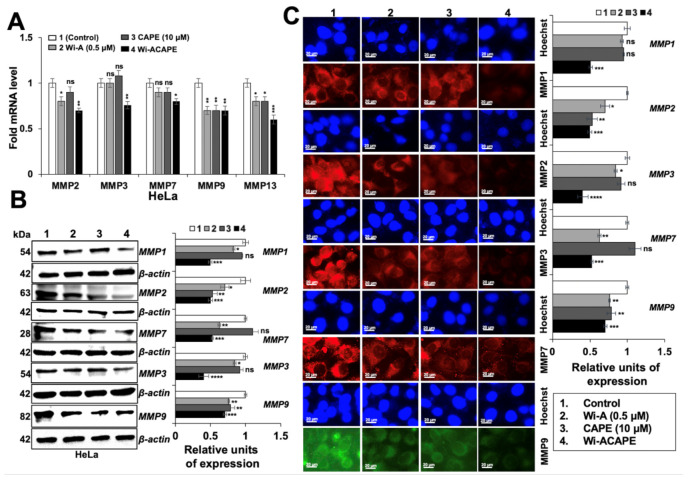
Wi-ACAPE caused downregulation of matrix-metalloproteinases (MMPs). RT-qPCR (**A**), Western blotting (**B**) and immunocytochemistry (**C**) analyses showed downregulation of MMP-1, -2, -3, -7 and -9 in Wi-ACAPE-treated (48 h) HeLa cells. Data were normalized against control and plotted as fold difference. Each data set represented the mean SD of at least three independent experiments. Statistical significance was defined as values of *p* > 0.05 (ns), *p* ≤ 0.05 (*), *p* ≤ 0.01 (**), *p* ≤ 0.001 (***) and *p* ≤ 0.0001 (****), which represent non-significant, significant, very significant, highly significant and extremely significant, respectively. The uncropped blots are shown in Figure S8.

Furthermore, mortalin and hnRNP-K, that are enriched in cancer cells and play a critical role in carcinogenesis and metastasis [14,66], showed a significant decrease in Wi-ACAPE treated cells. These findings were also endorsed by immunocytochemistry analyses (Figure 4B). mRNA analysis of these proteins in control and treated cells showed similar results, supporting that these changes occur at the transcript level (Figure 4C). In view of these data, we next examined the expression of Matrix metalloproteinases (MMPs) that play a critical role in cell migration, invasion and angiogenesis. As shown in Figure 5A, Wi-ACAPE caused stronger downregulation of MMP2, MMP3, MMP7 and MMP13 mRNA as compared to either of these compounds alone. MMP9 mRNA did not show any enhanced effect of Wi-ACAPE as compared to the individual compounds. Protein analyses by Western blotting and immunostaining showed a similar enhanced effect of Wi-ACAPE as compared to Wi-A/CAPE on MMP1, MMP2, MMP3, MMP7 and MMP9 proteins (Figure 5B,C and Figure S3B,D,F) suggesting that Wi-ACAPE causes reversal of EMT signaling.

### 3.7. Wi-ACAPE Treated Cells Showed Inactivation of VEGF Signaling

**Figure 6:**
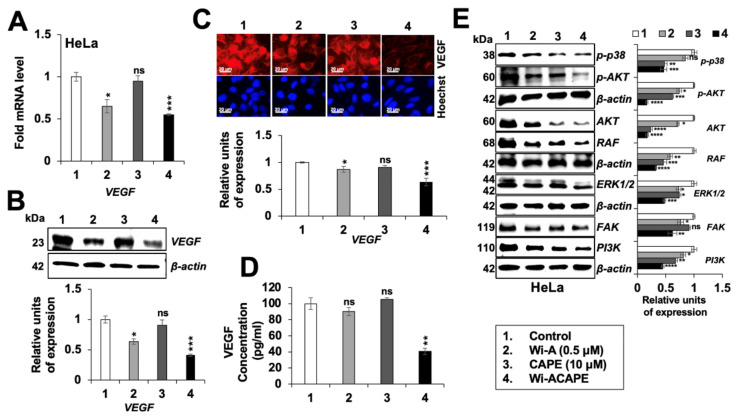
Wi-ACAPE caused inactivation of VEGF-signaling. Wi-ACAPE-treated (48 h) HeLa cells showed downregulation of VEGF mRNA (**A**) and protein ((**B**–**D**), as detected by Western blotting, Immunostaining and ELISA analyses, respectively). Several VEGF-driven effector proteins involved in metastasis and angiogenesis also showed a decrease in Wi-ACAPE-treated cells (**E**). Data were normalized against control and plotted as fold difference. Each data set represented the mean SD of at least three independent experiments. Statistical significance was defined as values of *p* > 0.05 (ns), *p* ≤ 0.05 (*), *p* ≤ 0.01 (**), *p* ≤ 0.001 (***) and *p* ≤ 0.0001 (****), which represent non-significant, significant, very significant, highly significant and extremely significant, respectively. The uncropped blots are shown in Figures S9 and S10.

VEGF is a key player in cancer cell metastasis and angiogenesis; in view of the cDNA array data and above analyses, we next examined the effect of Wi-A/CAPE/Wi-ACAPE on VEGF signaling. As shown in Figure 6A, VEGF mRNA was significantly downregulated in the Wi-A/Wi-ACAPE-treated cells; CAPE alone did not affect VEGF mRNA. Consistent with these data, VEGF protein showed a significant decrease in response to Wi-A/Wi-ACAPE treatment (Figure 6B,C, and Figure S3B,D,F). The data were endorsed by VEGF ELISA assay that showed a remarkable decrease in Wi-ACAPE-treated cells (Figure 6D). Furthermore, several VEGF-driven effector proteins such as p-p38MAPK (phospho-p38 mitogen-activated protein kinase), p-AKT (phospho-protein kinase B), FAK (focal adhesion kinase), ERK½ (extracellular-signal-regulated kinase), PI3K (phosphatidyl inositol-3-kinase) and RAF involved in metastasis and angiogenesis showed significant downregulation (Figure 6E). These results were also in line with the cDNA array result and RT-qPCR result (Figure S3G). In each case, Wi-ACAPE-treated cells showed stronger downregulation as compared to the cells treated with either Wi-A or CAPE (Figure 6), endorsing its higher potency for anti-metastatic activity.

**Figure 7:**
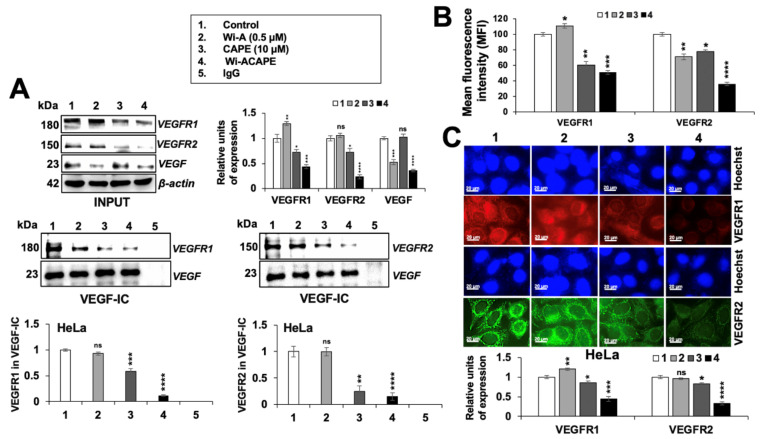
Wi-ACAPE blocked the interactions of VEGF to its receptors. Wi-ACAPE-treated (48 h) HeLa cells showed inhibition of VEGF-VEGFR1/R2 interactions. Immunoprecipitation of VEGF showed co-immunoprecipitation of VEGFR1 and VEGFR2 in control cells, and a decrease in Wi-A, CAPE and Wi-ACAPE treated cells. The latter showed a maximum decrease (**A**). Downregulation of cell surface expression of VEGFR1 and VEGFR2 was detected in Wi-ACAPE-treated HeLa cells through flow cytometry (**B**). Immunocytochemistry showed a decrease in VEGFR1 and VEGFR2 (**C**) analysis. Data were normalized against control and plotted as fold difference. Each data set represented the mean SD of at least three independent experiments. Statistical significance was defined as values of *p* > 0.05 (ns), *p* ≤ 0.05 (*), *p* ≤ 0.01 (**), *p* ≤ 0.001 (***) and *p* ≤ 0.0001 (****), which represent non-significant, significant, very significant, highly significant and extremely significant, respectively. The uncropped blots are shown in Figure S11.

Since VEGF signaling is dependent on the interaction of VEGF with VEGF receptors (VEGFR1/VEGFR2) [67], we next determined the effect of Wi-A/CAPE/Wi-ACAPE on VEGF-VEGFR complex by co-immunoprecipitation analyses. Anti-VEGF mouse monoclonal antibody was used to immunoprecipitated VEGF-VEGFR complex from control and treated cell lysates. The amount of VEGFR in the complex was detected by Western blotting of the complex with anti-VEGFR1 and anti-VEGFR2 antibodies. As shown in Figure 7A, complexes from Wi-ACAPE-treated cells showed a significant decrease in VEGF-VEGFR1 and VEGF-VEGFR2 complexes. On the other hand, Wi-A did not cause any change for VEGF complex with either VEGFR1 or VEGFR2, and CAPE caused a decrease in both. Of note, Wi-ACAPE-treated HeLa cells showed a decrease in VEGF, VEGFR1 and VEGFR2 levels (Figure 7A, input lanes and 7B). We also investigated cell surface expression of VEGFR1 and VEGFR2 expression in control and treated cells by flow cytometry (FACS). As shown in Figure 7C, a sharp decrease in the level of VEGFR1 as well as VEGFR2 expression was detected in Wi-ACAPE-, as compared to Wi-A/CAPE-, treated cells (Figure 7C).

## 4. Discussion

We had earlier developed a combination of Wi-A and CAPE that showed higher anticancer potential in vitro and in vivo [36]. Of note, the most common cause of cancer-related deaths is linked to the cancer metastasis supported by the acquisition of migration and invasion capacities, and the development of a new vascular network (angiogenesis) that is essential for the establishment of tumors at primary and secondary sites in the body [14,68]. In view of cancer therapy and prevention of relapse, low nontoxic doses of the anticancer compounds are considered important. In this regard, validation of anti-migration and anti-invasion activities is extremely important [69,70]. Cell adhesion molecules, Wnt/β-catenin signaling and vascular endothelial growth factor (VEGF) have been established as the major signaling pathways that regulate tumor metastasis and angiogenesis through Epithelial to Mesenchymal Transition (EMT) [60,71,72,73]. Interfering with these signaling molecules and pathways through natural compounds has been shown to inhibit metastasis, angiogenesis and cancer stemness at in vitro and in vivo levels [74]. Wi-A, the active anticancer compound of Ashwagandha, has been shown to possess anti-inflammatory, anti-proliferation, anti-metastatic and anti-angiogenic properties, suggesting its potential as an anticancer drug [37,41,75,76,77,78,79]. Wi-A and its combination with Withanone (Wi-N) [78,80], cisplatin [81] and paclitaxel [37] were shown to have superior effects in terms of tumor suppression, anti-metastasis, overcoming drug resistance and preventing tumor relapse. Similarly, the combination of Wi-N and Cucurbitacin B was shown to induce senescence in cancer cells, selectively [74]. Caffeic Acid Phenethyl Ester (CAPE)—an anticancer ingredient derived from honeybee propolis—has been shown to have anti-metastatic and anti-angiogenic activities through its multiple modes of action [82,83,84,85,86]. Using human ovarian and cervical cancer cells, we previously reported that a combination of Wi-A and CAPE (1 µM and 20 µM) demonstrated a better anticancer potential as compared to each of the two compounds alone [36]. These studies have confirmed that a combinational approach to cancer treatment may be a safer and more effective option. In this study, we investigated the possible molecular roles of Wi-A, CAPE and their combination: Wi-ACAPE (0.5 µM, 10 µM or 0.5 µM + 10 µM), in suppressing metastasis and angiogenesis. We used HeLa cells as an in vitro model for cell-based and molecular assays. Firstly, we found that Wi-ACAPE enhanced the inhibition of migration, invasion and angiogenesis by dysregulation of several signaling pathways (cell adhesion molecules, tight junction, MAPK and VEGF signaling) that are critically involved in these phenotypes. Wi-ACAPE showed a dysregulation of the E-cadherin/β-catenin signaling as indicated by the increased expression of E-cadherin and the decreased expression of β-catenin (Figure 3).

E-cadherin, a 120-kDa transmembrane glycoprotein, is critically engaged in cancer invasion and metastasis through the EMT process. The idea of restoring the E-cadherin expression has been considered as viable to inhibit its downstream oncogenic EMT signaling [87]. Several natural compounds have been reported to suppress cell migration, invasion and angiogenesis in vitro through the upregulation of E-cadherin [24,88,89,90,91,92]. We report that a unique combination of Wi-A and CAPE (Wi-ACAPE) causes the strong stimulation of E-cadherin expression and reversal of EMT as supported by inhibition of the Wnt/β-catenin, MMP and VEGF signaling pathways. Of note, a decrease in nuclear β-catenin in the Wi-ACAPE treated cells was associated with a decrease in Wnt/β-catenin regulated genes (*c-Myc, Cyclin D1* and *AXIN*) critically involved in cancer metastasis (Figure 3). Consistent with these data, Wi-ACAPE-treated cells showed a strong reduction in mesenchymal proteins (fibronectin, vimentin, N-cadherin and MMPs) (Figure 4 and Figure 5).

It has been reported that stress proteins mortalin/mthsp70 and CARF (the Collaborator of ARF) regulate EMT in cancer cells. Both of these proteins are enriched in cancer cells and have been shown to contribute to cell migration and EMT signaling pathways (PI3K-Akt, JAK–STAT and focal adhesion signaling) [14,15,18,30,93,94,95,96,97,98]. Wi-ACAPE caused downregulation of both mortalin and CARF (Figure 3 and Figure 4), supporting multimodal action of Wi-ACAPE. Furthermore, VEGF signaling—a key driver of angiogenesis—is considered as a therapeutic target for most cancer types. Since its activity is regulated by binding to its receptors (VEGFR1 and VEGFR2) possessing tyrosine kinase activity, several approaches including VEGF blockades, VEGF-trap and tyrosine kinase inhibitors [2,31] have been validated in laboratory and clinical studies. Bevacizumab, the FDA-approved VEGF-targeted drug, has been shown to downregulate VEGF as well as inhibit its interaction with VEGFRs [99]. However, adverse events associated with bevacizumab remain a concern [100] warranting the development of a new class of safer drugs. In this study, Wi-ACAPE inhibited the migration, invasion and angiogenic potential of HeLa cells by downregulation of not only VEGF mRNA and protein, but also its receptors. Furthermore, binding of VEGF to its receptors was also blocked. Downregulation of VEGF resulting in Wi-ACAPE-treated cells was endorsed by decrease in p38MAPK, AKT, FAK, ERK, PI3K and RAF proteins signifying effective inhibition of metastatic and angiogenic signaling (Figure 6). Taken together with our earlier findings that a combination of Wi-A and CAPE caused selective cytotoxicity in cancer cells through the modulation of p53-mortalin and PARP1 signaling [36], we report that a low dose combination of Wi-A and CAPE (Wi-ACAPE) possesses significant anti-metastasis and anti-angiogenic potentials, warranting further experimental and clinical trials.

## 5. Conclusions

We demonstrate that a low dose combination of Wi-A and CAPE (Wi-ACAPE) causes reversal of EMT by the upregulation of E-cadherin and Claudin1, yielding inhibition of Wnt/β-catenin, Vimentin, MMPs, VEGF and VEGFR signaling pathways. The data suggest the potential of Wi-ACAPE for the treatment of metastatic cancers and, hence, warrants further studies on validation of its efficacy in in vivo and clinical trials.

## Data Availability

All datasets used and/or analyzed during the current study are included in the manuscript and Supplementary Information Files.

## Supplementary Materials

The following supporting information can be downloaded at: https://www.mdpi.com/article/10.3390/cancers14030787/s1, Figure S1: Wi-A/CAPE/Wi-ACAPE (at the indicated doses) were not cytotoxic neither to human cervical/breast cancer cells nor to normal lung fibroblasts cells. Figure S2: Low dose of Wi-A/CAPE/Wi-ACAPE did not cause activation of apoptosis signaling. Figure S3: Wi-ACAPE showed anti-metastatic potential in breast cancer in vitro. Figure S4: Effect of Withaferin A and CAPE (Wi-ACAPE (High)) in combination on metastasis protein markers. Figure S5: Full uncropped Western blots of proteins of interest (Claudin1, E-cadherin and β-catenin) detected from both E-cadherin immunocomplexes and input detected from Wi-A/CAPE/Wi-ACAPE-treated and control HeLa cell lysates (Figure 2C and Figure 3A,C). Figure S6: Full uncropped Western blots for the proteins of interest (CARF, Wnt, c-Myc and Cyclin D1) detected from Wi-A/CAPE/Wi-ACAPE-treated and control HeLa cell lysates (Figure 3F). Figure S7: Full uncropped Western blots for the proteins of interest (Mortalin, Fibronectin, Vimentin, N-cadherin, and hnRNP-K) detected from Wi-A/CAPE/Wi-ACAPE-treated and control HeLa cell lysates (Figure 4A). Figure S8: Full uncropped Western blots for the proteins of interest (MMP1, MMP2, MMP7, MMP3 and MMP9) detected from Wi-A/CAPE/Wi-ACAPE-treated and control HeLa cell lysates (Figure 5B). Figure S9: Full uncropped Western blots for the proteins of interest (VEGF) detected from Wi-A/CAPE/Wi-ACAPE-treated and control HeLa cell lysates (Figure 6B). Figure S10: Full uncropped Western blots for the proteins of interest (p-p38, p-AKT, AKT, RAF, ERK1/2, FAK, PI3K) detected from Wi-A/CAPE/Wi-ACAPE-treated and control HeLa cell lysates (Figure 6E). Figure S11: Full uncropped Western blots for the protein of interest (VEGFR1 and VEGFR2) detected from both VEGF immunocomplexes and input of Wi-A/CAPE/Wi-ACAPE-treated and control HeLa cell lysates (Figure 7A). Figure S12: Full uncropped Western blots for the proteins of interest (p53, PARP-1, Bcl-2) and (MMP3, vimentin) detected from Wi-A/CAPE/Wi-ACAPE-treated and control HeLa and TIG-3 cells lysates, respectively. β-actin was used as an internal loading control. Figure S13: Full uncropped Western blots for the proteins of interest (Claudin-1, MMP3, E-cadherin, β-catenin, Vimentin, VEGF) detected from Wi-A/CAPE/Wi-ACAPE-treated and control MCF-7 cell lysates. β-actin was used as an internal loading control. Figure S14: Full uncropped Western blots for the proteins of interest (Claudin-1, MMP3, E-cadherin, β-catenin, Vimentin, VEGF) detected from Wi-A/CAPE/Wi-ACAPE-treated and control Mot-OE MCF-7 cell lysates. β-actin was used as an internal loading control. Figure S15: Full uncropped Western blots for the proteins of interest (Claudin-1, MMP3, E-cadherin, β-catenin, Vimentin, VEGF) detected from Wi-A/CAPE/Wi-ACAPE-treated and control MDA-MB-231 cell lysates. β-actin was used as an internal loading control. Figure S16: Full uncropped Western blots for the proteins of interest (Claudin1, E-cadherin, MMP3, Vimentin) detected from Wi-A/CAPE/Wi-ACAPE-treated and control HeLa cell lysates. β-actin was used as an internal loading control. Table S1: Primer sequences used for Real Time quantitative Polymerase Chain Reaction (RT-qPCR).
