# Supplementary material for: A Low Dose Combination of Withaferin A and Caffeic Acid Phenethyl Ester Possesses Anti-Metastatic Potential In Vitro: Molecular Targets and Mechanisms
Source: Cancers (Basel). 2022 Feb 3;14(3):787. doi: 10.3390/cancers14030787 (PMC8834371; doi:10.3390/cancers14030787)
Supplement: Supplementary file 1 [file cancers-14-00787-s001.zip › cancers-1523562-supplementary.pdf]

Supplementary Materials: A Low Dose Combination of Withaferin A and Caffeic Acid Phenethyl Ester Possesses Anti-Metastatic Potential In Vitro*:* Molecular Targets and Mechanisms

Anissa Nofita Sari, Jaspreet Kaur Dhanjal, Ahmed Elwakeel, Vipul Kumar, Hazna Noor Meidinna, Huayue Zhang, Yoshiyuki Ishida, Keiji Terao, Durai Sundar, Sunil C. Kaul and Renu Wadhwa

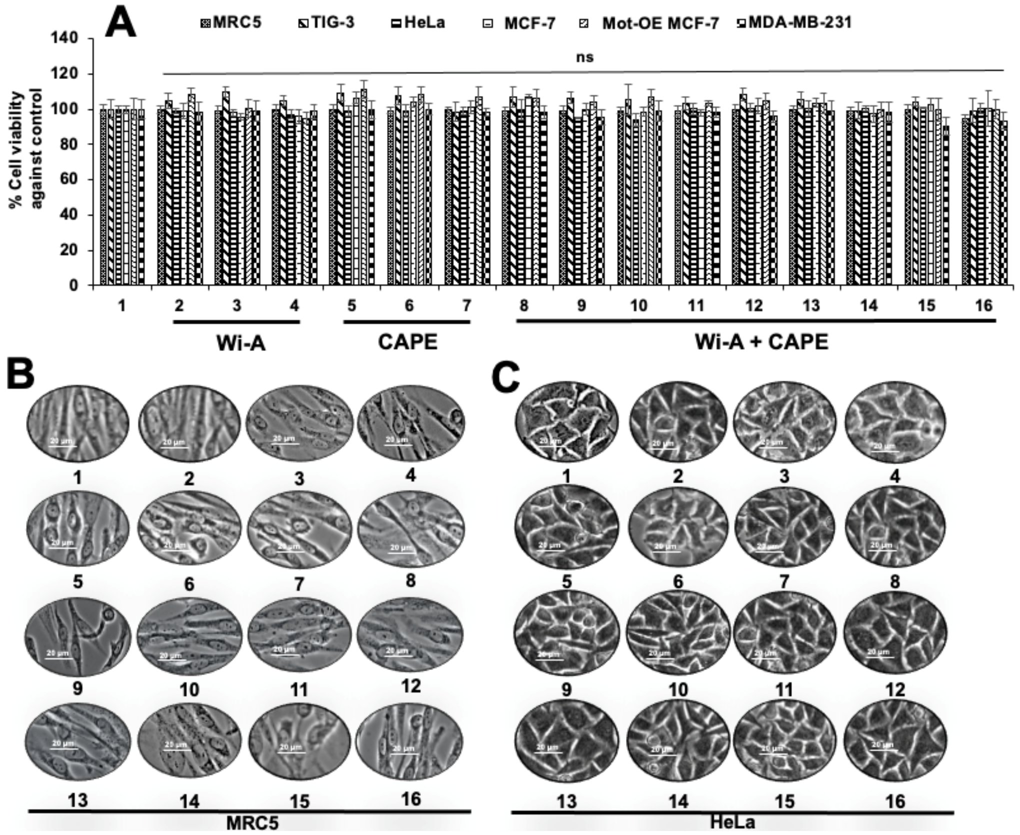

	Compounds	
	Wi-A (µM)	CAPE (µM)
1	0	0
2	0.05	0
3	0.1	0
4	0.5	0
5	0	1
6	0	5
7	0	10
8	0.05	1
9	0.05	5
10	0.05	10
11	0.1	1
12	0.1	5
13	0.1	10
14	0.5	1
15	0.5	5
16	0.5	10

Figure S1. Wi-A/CAPE/Wi-ACAPE (at the indicated doses) were cytotoxic neither to human cervical/breast cancer cells nor to normal lung fibroblasts cells. Short-term (48 h) cell-viability assays for control, Wi-A/CAPE/Wi-ACAPE-treated cells (MRC5 and TIG-3 lung fibroblasts; HeLa cervical cancer; MCF-7, Mot-OE MCF-7 and MDA-MB-231 breast cancer cells) (**A**). Phase contrast micrographs of Wi-A/CAPE/Wi-ACAPE-treated cells did not reveal any stress phenotype (condensation and blebbing morphologies) as compared to the untreated control (**B** and **C**). The quantified cell-viability data represents mean ± SD obtained from three independent biological replicates; *p*-values were calculated using unpaired Student's *t*-test. $p > 0.05$ (^{ns}) represent non-significant.

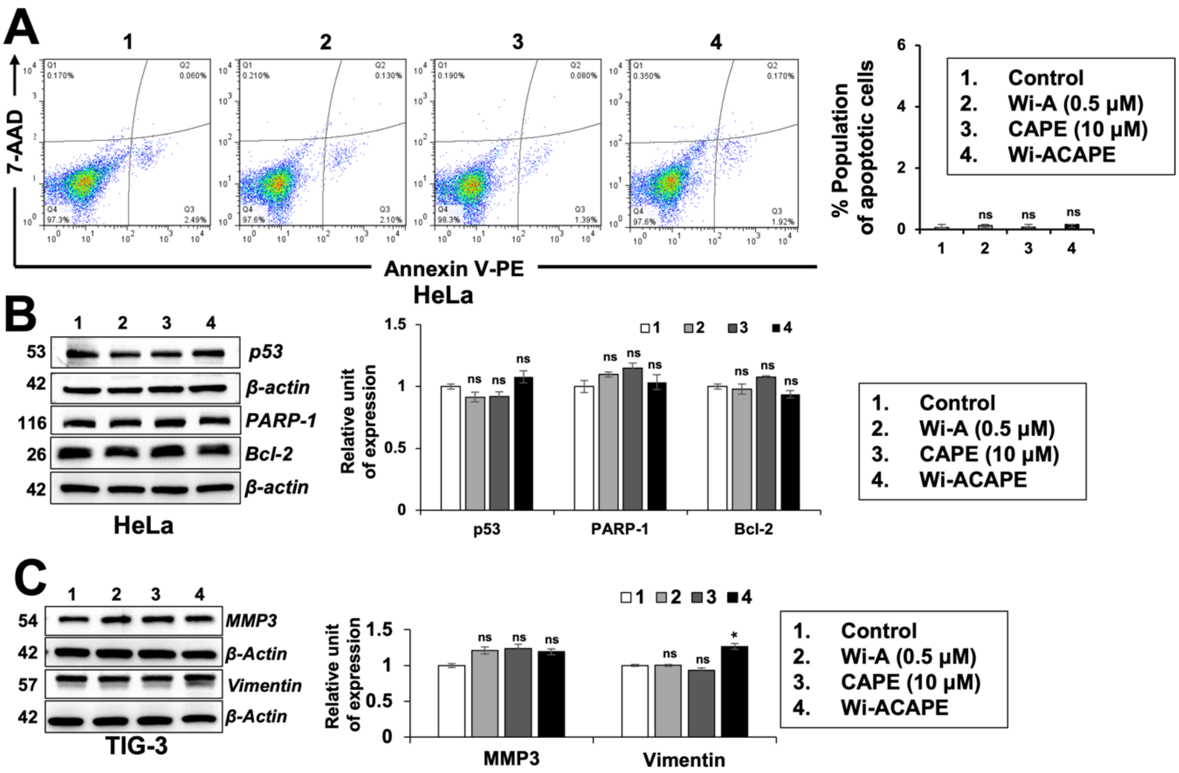

Figure S2. Low dose of Wi-A/CAPE/Wi-ACAPE did not cause activation of apoptosis signaling. Flow cytometric analysis showed the absence of apoptotic cells when treated with Wi-A (0.5 µM), CAPE (10 µM), or their combination for 48 h (**A**). Western blot analysis of Wi-A, CAPE, and their combination treated cells showing no significant effect on PARP1, p53, and Bcl2 expressions (**B**). Wi-ACAPE-treated (48 h) TIG-3 cells did nor caused downregulation of MMP3 and Vimentin protein as detected by Western blotting (**C**). Each data set represented the mean SD of at least three independent experiments. Statistical significance was defined as values of $p > 0.05$ (ns) that represent non-significant. The uncropped blots are shown in Figure S12.

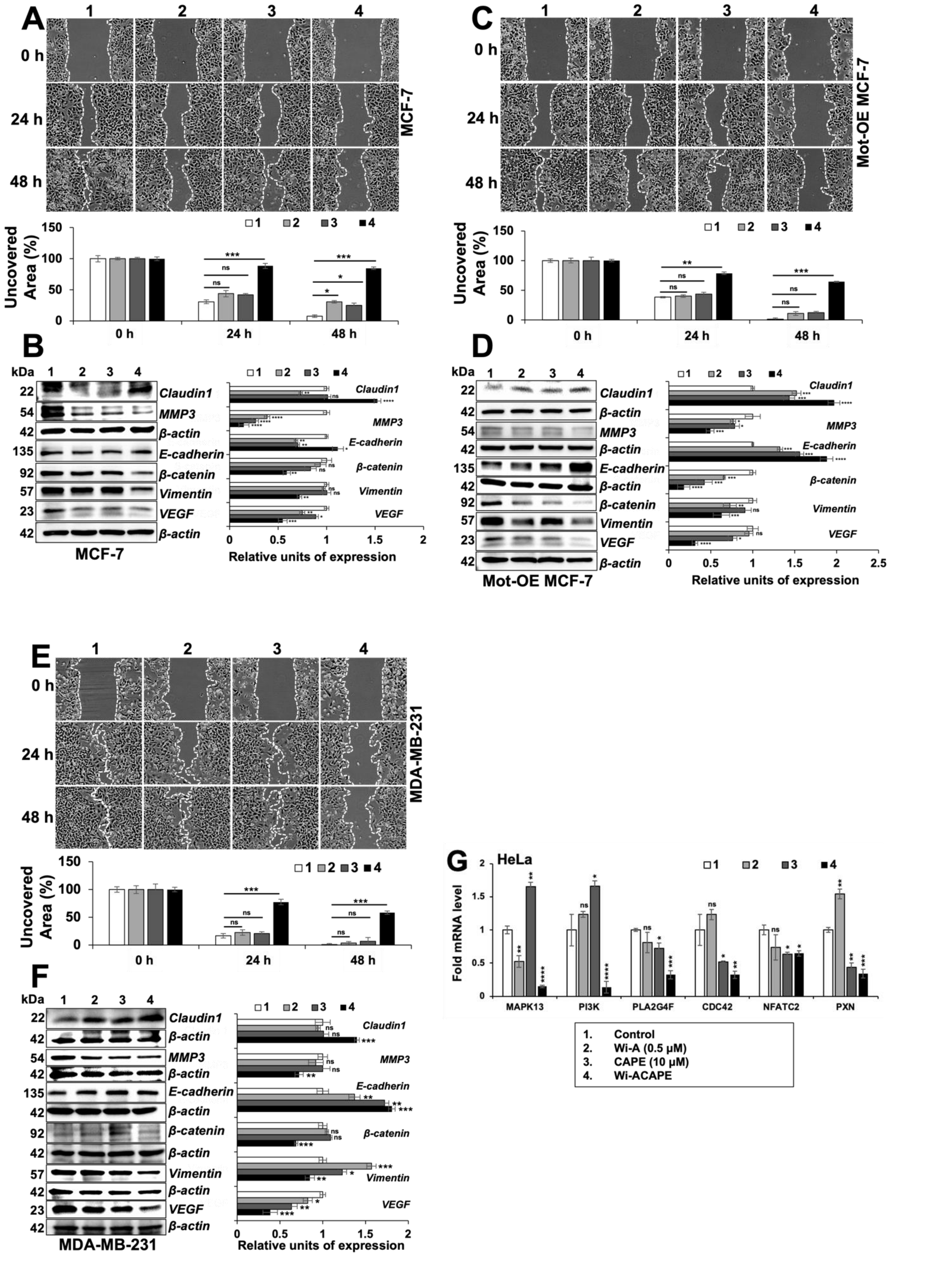

Figure S3. Wi-ACAPE showed anti-metastatic potential in breast cancer in vitro. The stronger effects on the delay of MCF-7, Mot-OE MCF-7, MDA-MB-231 cells migration were observed in 48 h treatment with Wi-ACAPE than with Wi-A or CAPE individually (**A**,**C**,**E**). Western blotting analyses showed upregulation of Claudin-1 and E-cadherin, and downregulation of MMP3, B-catenin,

Vimentin, VEGF after treatment of Wi-ACAPE for 48 h (**B**,**D**,**F**). Wi-A/CAPE/Wi-ACAPE downregulated the mRNA expressions of several downstream effectors of VEGF-VEGFR signaling (**G**). Data were normalized against control and plotted as fold difference. Each data set represented the mean SD of at least three independent experiments. Statistical significance was defined as values of $p >$ 0.05 (ns), $p \leq 0.05$ (*), $p \leq 0.01$ (**), $p \leq 0.001$ (***), and $p \leq 0.0001$ (****), which represent non-significant, significant, very significant, highly significant, and extremely significant, respectively. The uncropped blots are shown in Figure S13, Figure S14 and Figure S15.

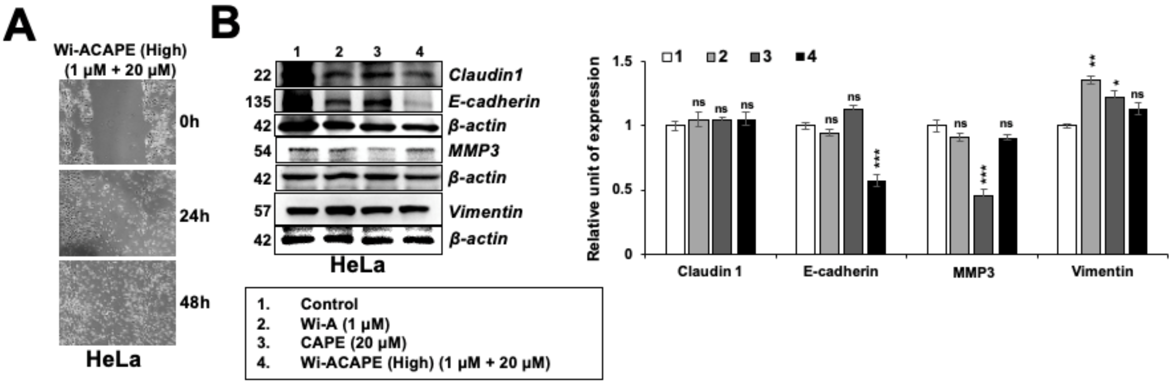

Figure S4. Effect of Withaferin A and CAPE in combination (Wi-ACAPE (High)) on metastasis protein markers. Wi-ACAPE (High) (1 µM + 20 µM) caused apoptosis in cells and did not delay the migration in HeLa cells (**A**). Western blotting analyses showed decrease in E-cadherin; Claudin1, MMP3 and Vimentin did not show any significant change in the HeLa cells after treatment with Combination of (1 µM Wi-A + 20 µM CAPE) (**B**). Statistical significance was defined as values of $p > 0.05$ (ns), $p \leq 0.05$ (*), $p \leq 0.01$ (**), and $p \leq 0.001$ (***) which represent non-significant, significant, very significant, and highly significant, respectively. The uncropped blots are shown in Figure S16.

Full uncropped Western blots presented in Figures 1–7 and Figures S2–S3

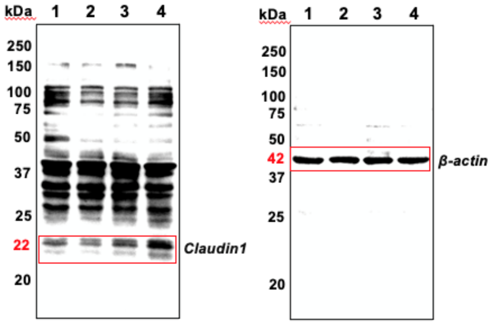

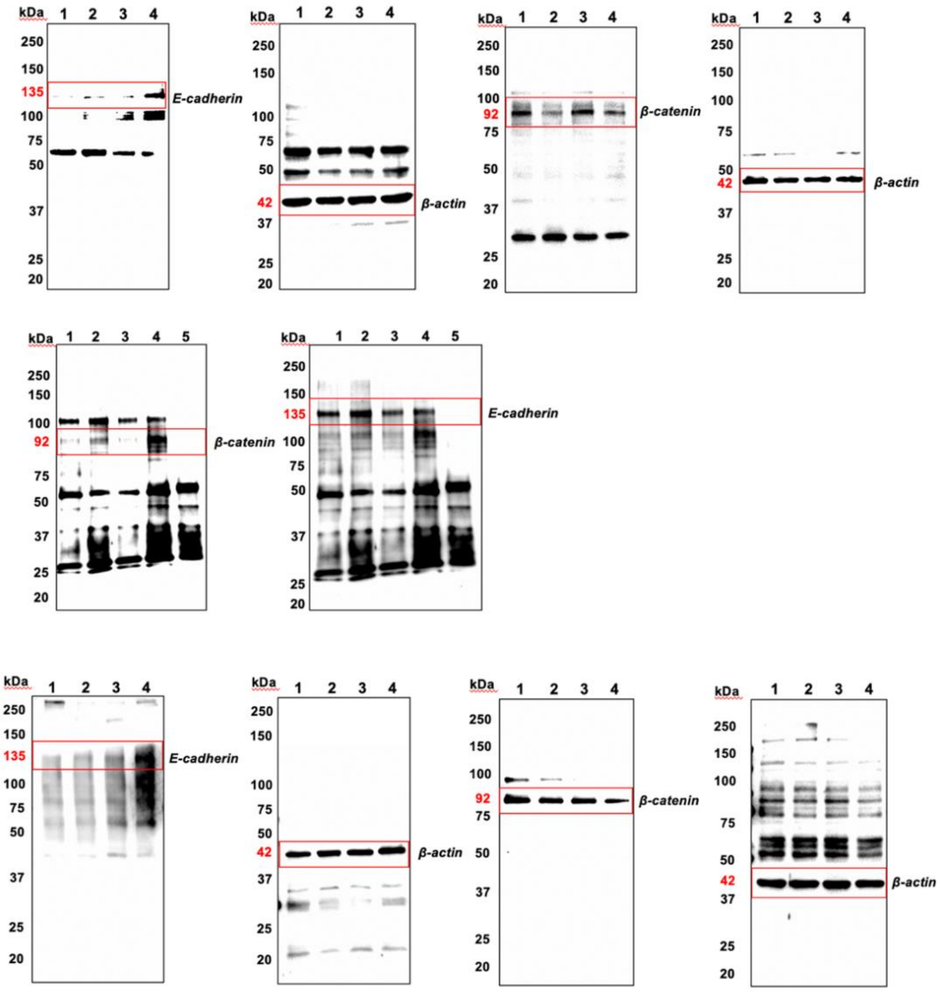

Figure S5. Full uncropped Western blots for the proteins of interest (Claudin1, E-cadherin and β-catenin) and β-catenin detected from both E-cadherin immunocomplexes and input of detected from Wi-A/CAPE/Wi-ACAPE-treated and control HeLa cell lysates (Figure 2C, Figure 3A and Figure 3C). For the immunoprecipitated samples, E-cadherin bands were used to normalize an equal immunocomplexes. For the input samples, β-actin was used as an internal loading control.

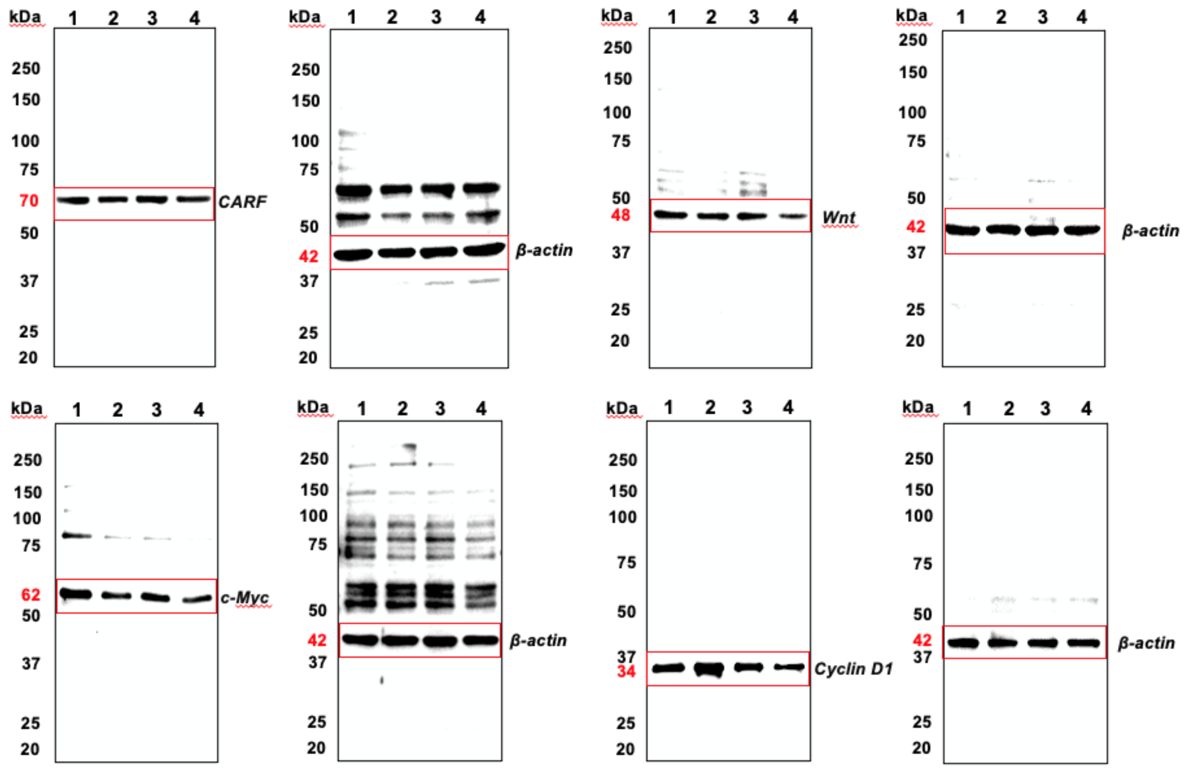

Figure S6. Full uncropped Western blots for the proteins of interest (CARF, Wnt, c-Myc and Cyclin D1) detected from Wi-A/CAPE/Wi-ACAPE-treated and control HeLa cell lysates (Figure 3F). β-actin was used as an internal loading control.

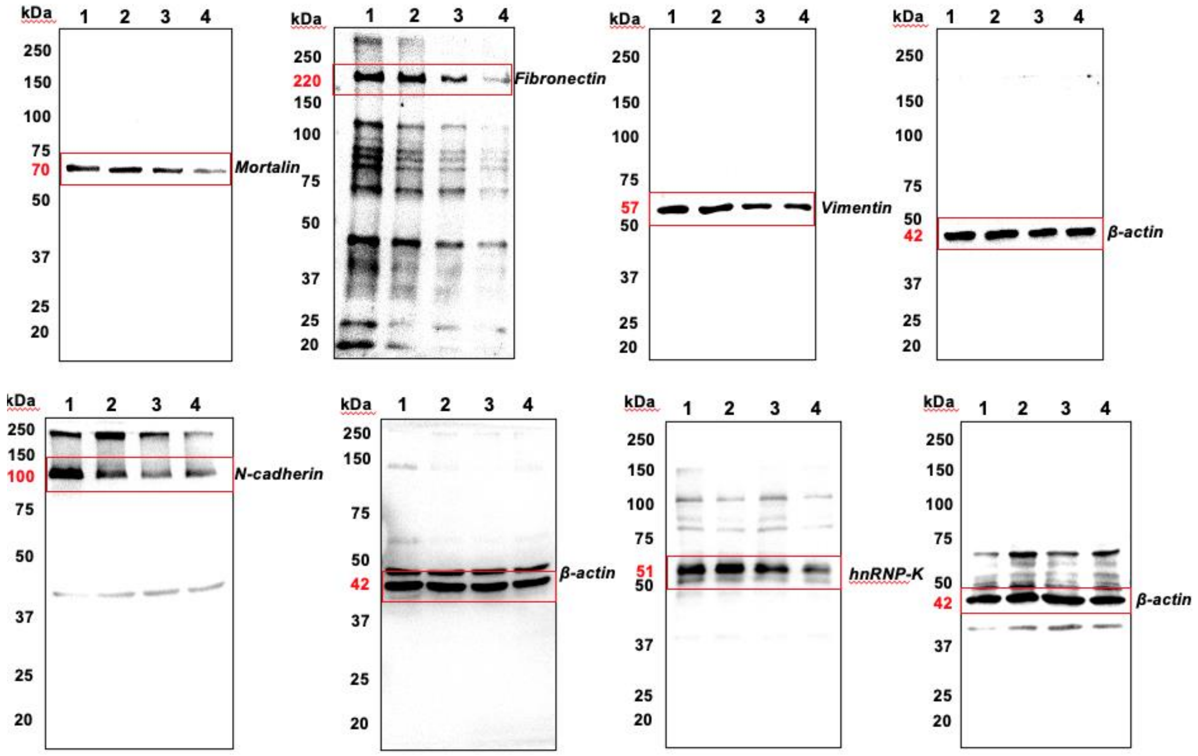

Figure S7. Full uncropped Western blots for the proteins of interest (Mortalin, Fibronectin, Vimentin, N-cadherin, and hnRNP-K) detected from Wi-A/CAPE/Wi-ACAPE-treated and control HeLa cell lysates (Figure 4A). β-actin was used as an internal loading control.

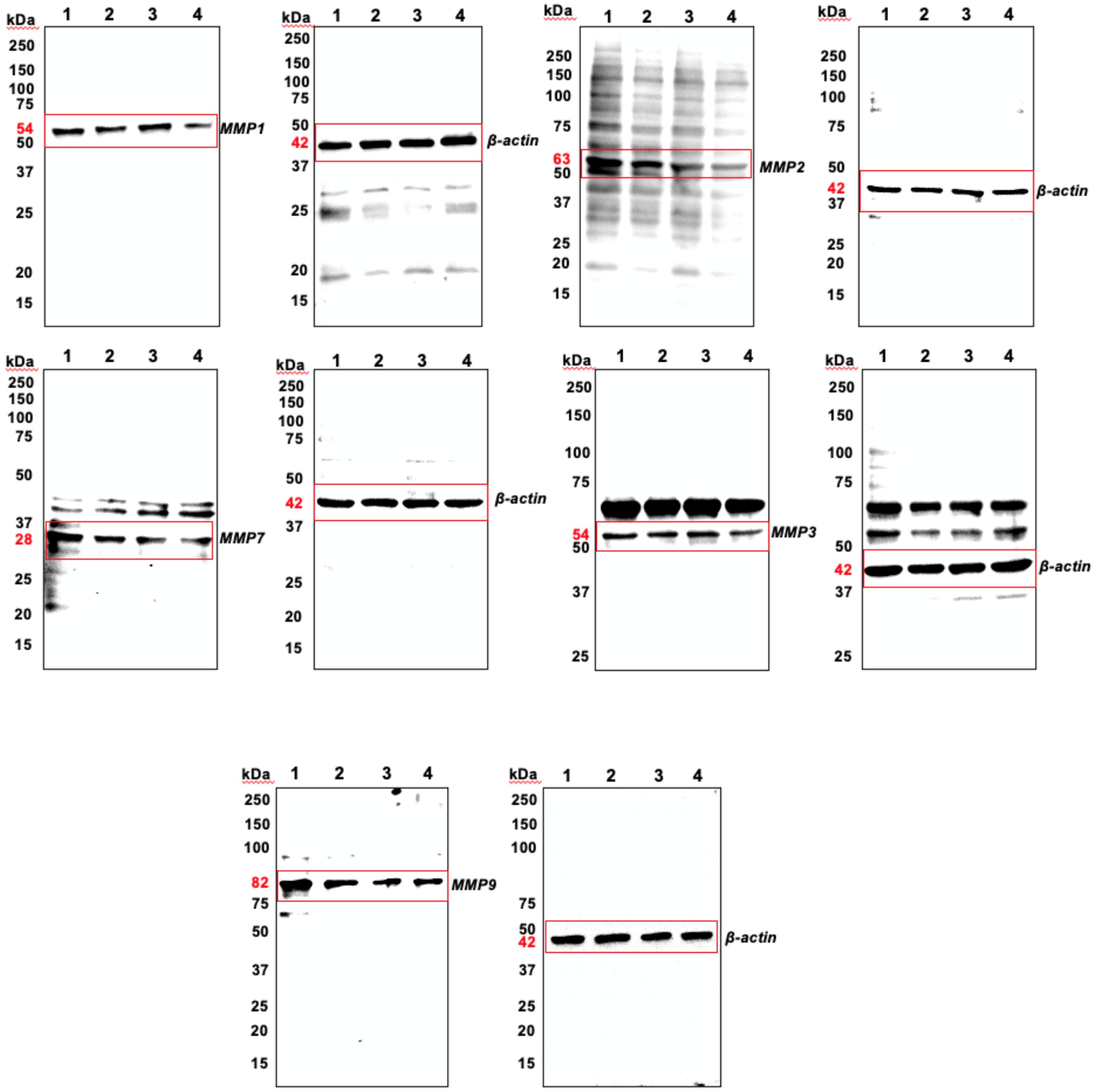

Figure S8. Full uncropped Western blots for the proteins of interest (MMP1, MMP2, MMP7, MMP3 and MMP9) detected from Wi-A/CAPE/Wi-ACAPE-treated and control HeLa cell lysates (Figure 5B). β-actin was used as an internal loading control.

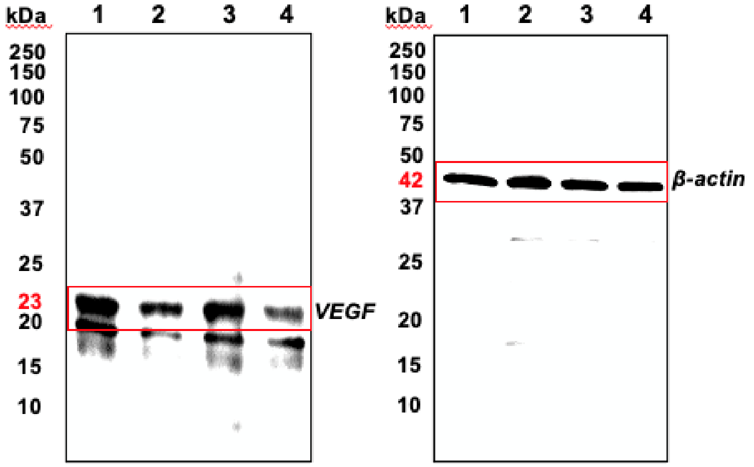

Figure S9. Full uncropped Western blots for the proteins of interest (VEGF) detected from Wi-A/CAPE/Wi-ACAPE-treated and control HeLa cell lysates (Figure 6B). β-actin was used as an internal loading control.

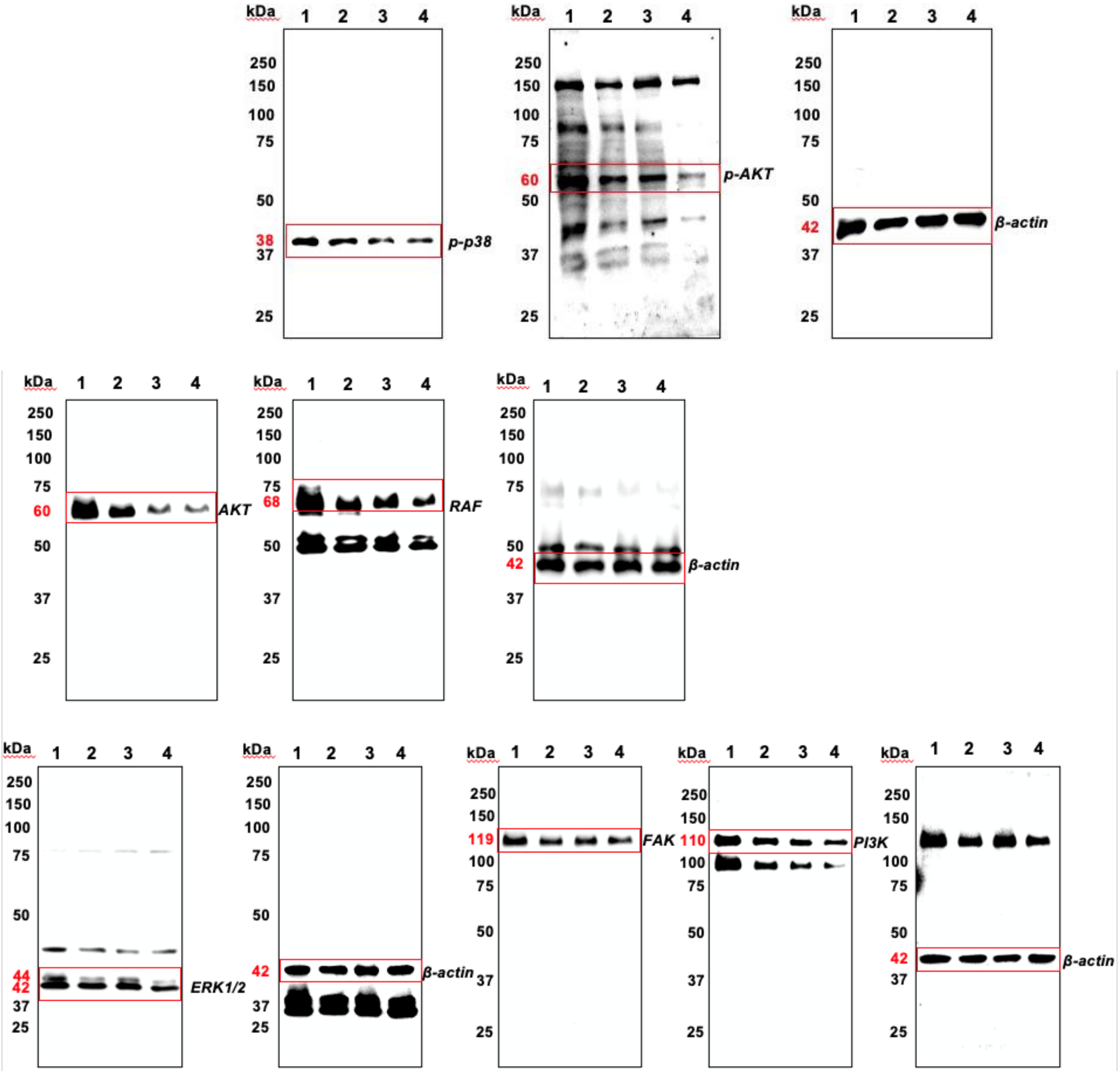

Figure S10. Full uncropped Western blots for the proteins of interest (p-p38, p-AKT, AKT, RAF, ERK1/2, FAK, PI3K) detected from Wi-A/CAPE/Wi-ACAPE-treated and control HeLa cell lysates (Figure 6E). β-actin was used as an internal loading control.

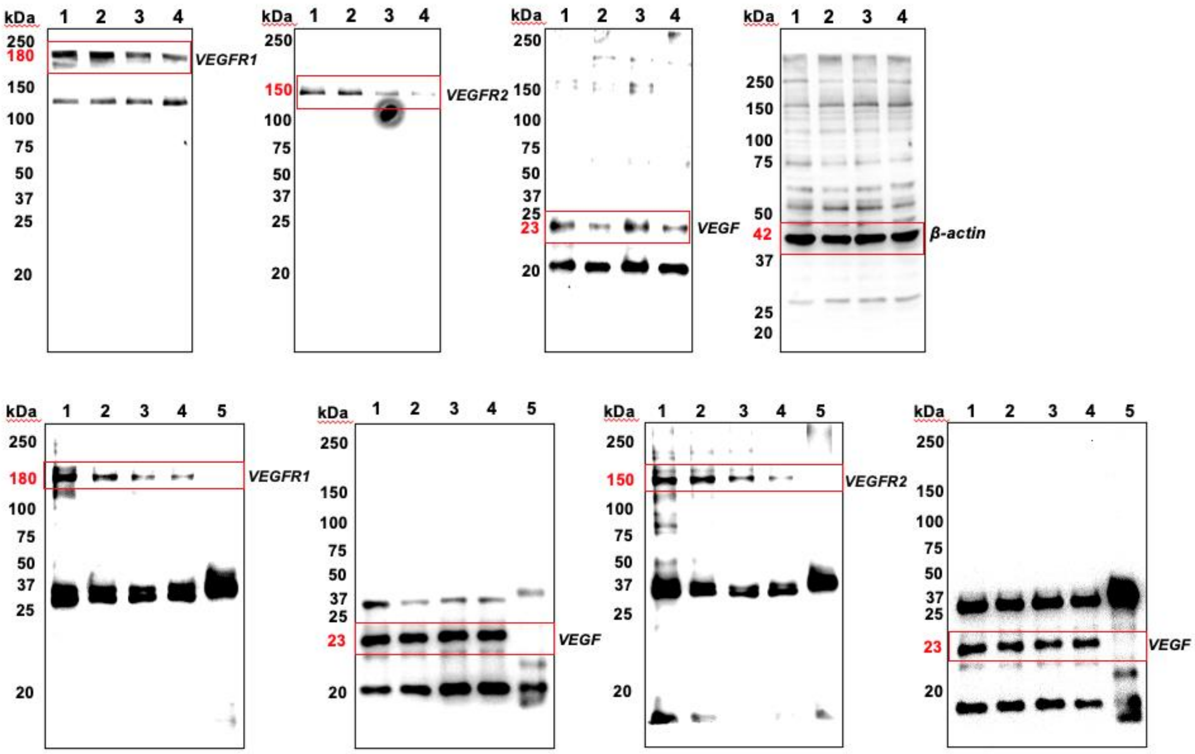

Figure S11. Full uncropped Western blots for the protein of interest (VEGFR1 and VEGFR2) detected from both VEGF immunocomplexes and input of Wi-A/CAPE/Wi-ACAPE-treated and control HeLa cell lysates (Figure 7A). For the immunoprecipitated samples, VEGF bands were used to normalize an equal immunocomplexes. For the input samples, β-actin was used as an internal loading control.

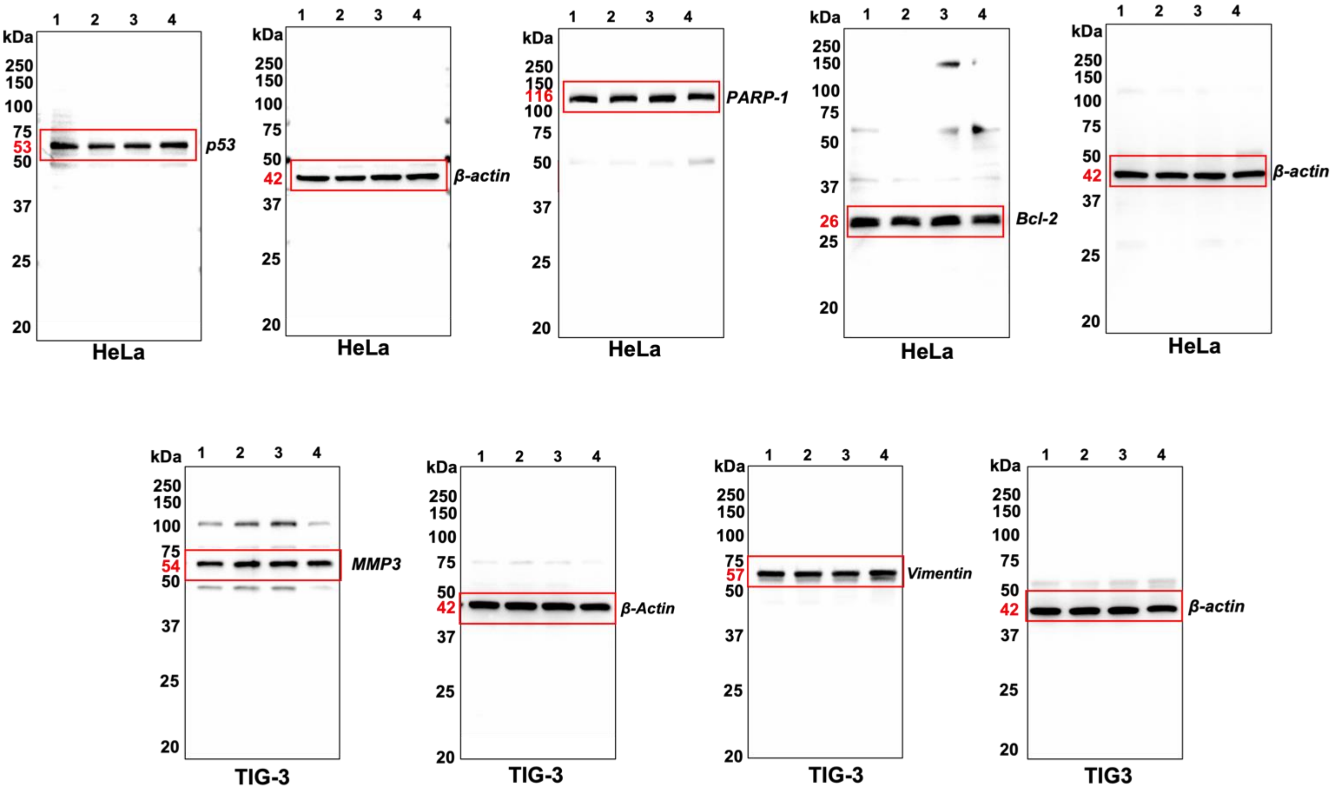

Figure S12. Full uncropped Western blots for the proteins of interest (p53, PARP-1, Bcl-2) and (MMP3, Vimentin) detected from Wi-A/CAPE/Wi-ACAPE-treated and control HeLa and TIG-3 cells lysates, respectively. β-actin was used as an internal loading control.

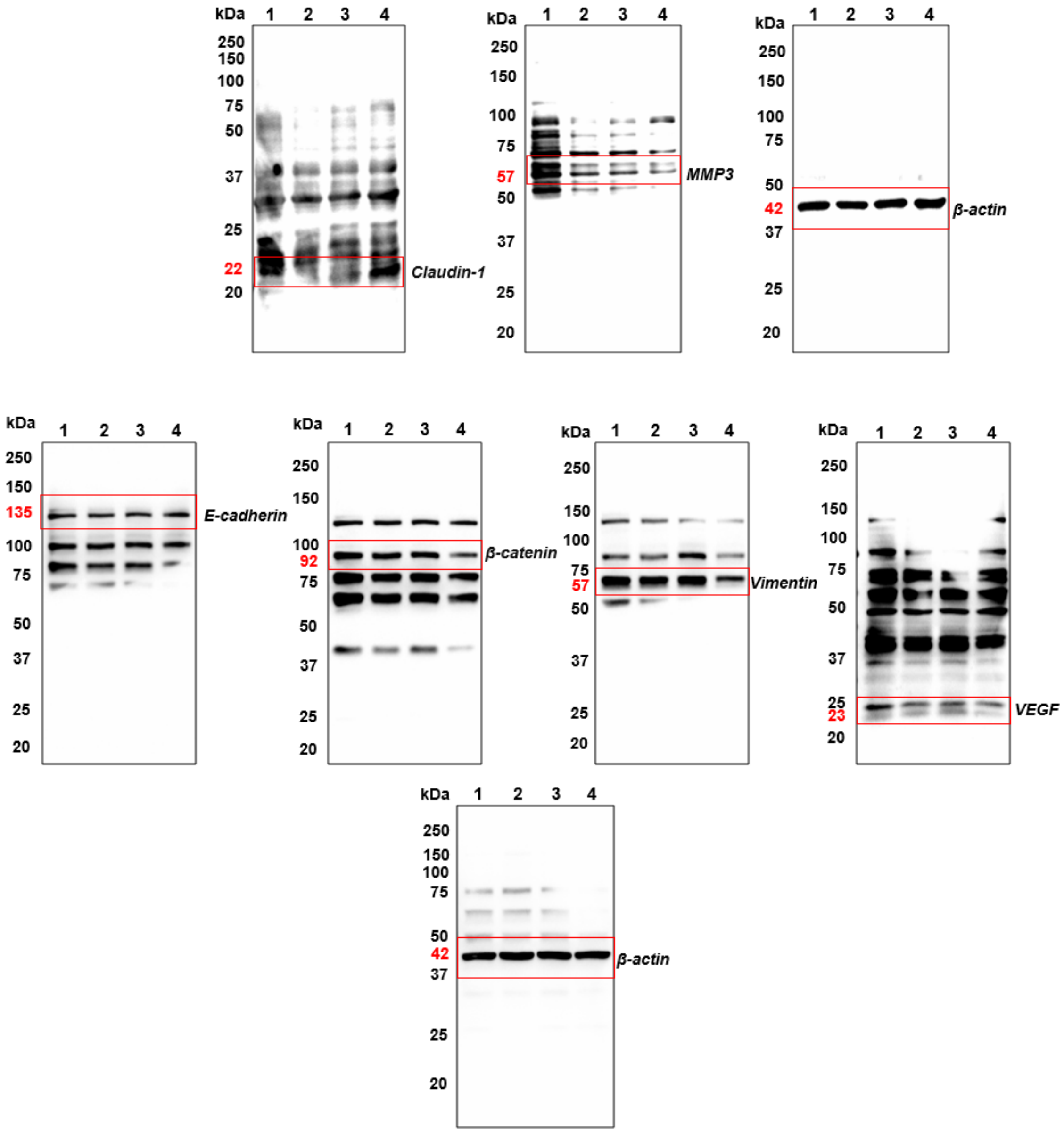

Figure S13. Full uncropped Western blots for the proteins of interest (Claudin-1, MMP3, E-cadherin, β-catenin, Vimentin, VEGF) detected from Wi-A/CAPE/Wi-ACAPE-treated and control MCF-7 cell lysates. β-actin was used as an internal loading control.

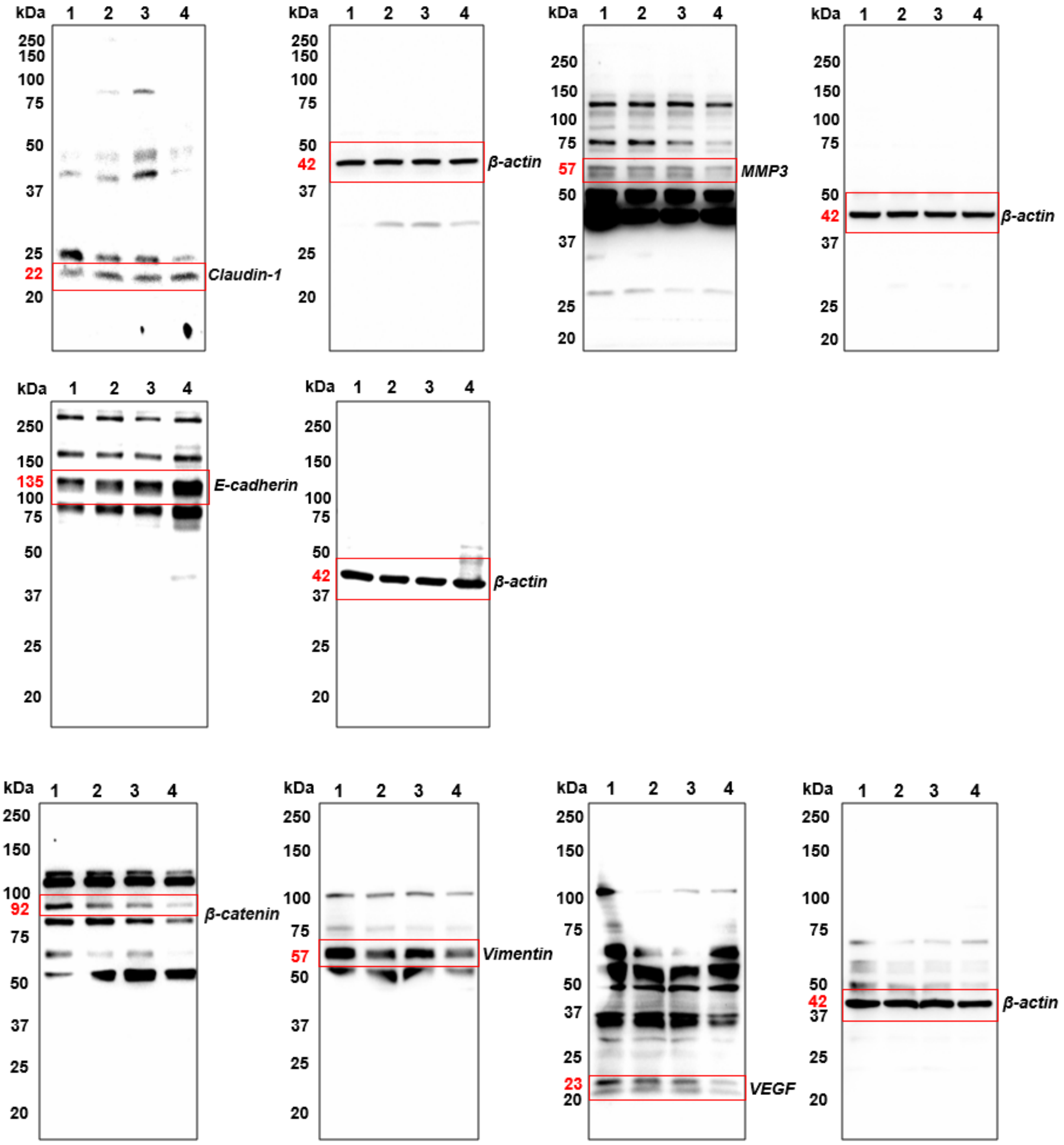

Figure S14. Full uncropped Western blots for the proteins of interest (Claudin-1, MMP3, E-cadherin, β-catenin, Vimentin, VEGF) detected from Wi-A/CAPE/Wi-ACAPE-treated and control Mot-OE MCF-7 cell lysates. β-actin was used as an internal loading control.

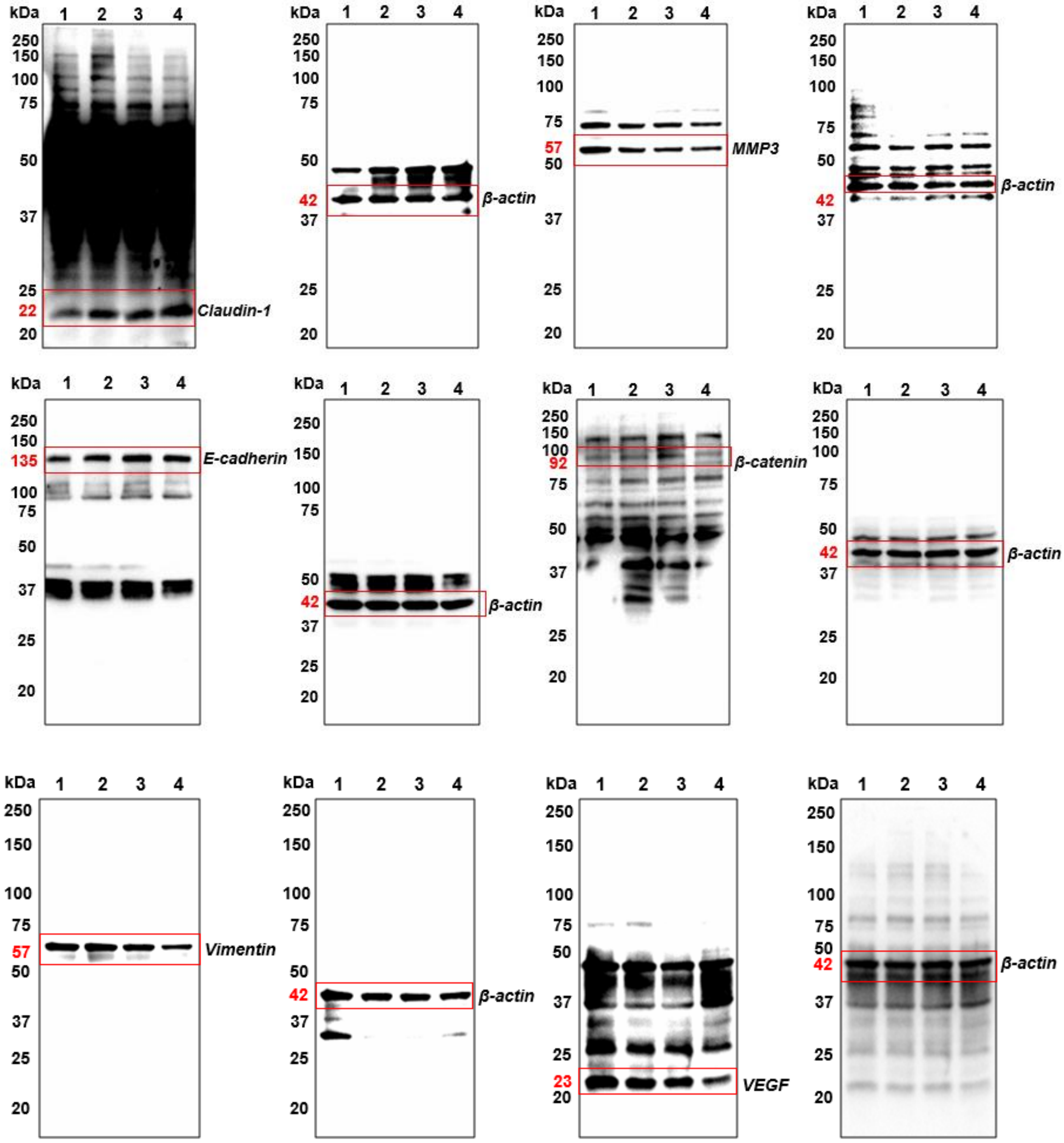

Figure S15. Full uncropped Western blots for the proteins of interest (Claudin-1, MMP3, E-cadherin, β-catenin, Vimentin, VEGF) detected from Wi-A/CAPE/Wi-ACAPE-treated and control MDA-MB-231 cell lysates. β-actin was used as an internal loading control.

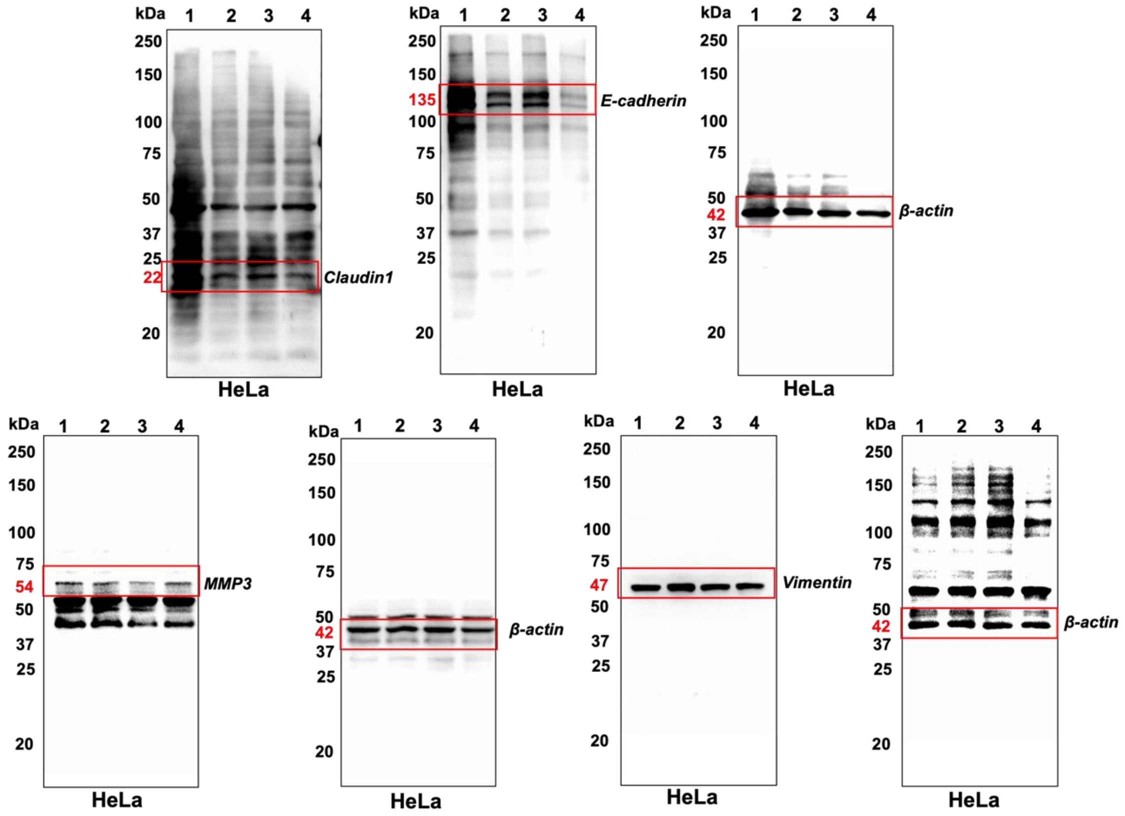

Figure S16. Full uncropped Western blots for the proteins of interest (Claudin1, E-cadherin, MMP3, Vimentin) detected from Wi-A/CAPE/Wi-ACAPE-treated and control HeLa cell lysates. β-actin was used as an internal loading control.

Table S1. Primer sequences used for Real Time quantitative Polymerase Chain Reaction (RT-qPCR).

Gene (Human)	Primer Sequence (5′–3′)
CLDN1 Forward	CAGCGAGTCATGGCCAAC
CLDN1 Reverse	TCTCAATGTCCATTTTCGGTTT
CLDN3 Forward	CGCGGCAGCCATGTCCAT
CLDN3 Reverse	GGTGGTGGTGGTGGGGTCTCC
CLDN6 Forward	AGAAGGATTCCAAGGCCCG
CLDN6 Reverse	GATGTTGAGTAGCGGGCCAT
CLDN14 Forward	CCAAGACCACCTTTGCCATCCT
CLDN14 Reverse	AGTTCTGCACCACGTCGTTGGT
OCLN Forward	ATGGCAAAGTGAATGACAAGCGG
OCLN Reverse	CTGTAACGAGGCTGCCTGAAGT
JAM2 Forward	GAACTGTGGTAGAGCTACGATGTC
JAM2 Reverse	TTTCACTCATTGTCGTGGCTTTAG
TJP2 Forward	ATTAGTGCGGGAGGATGCCGTT
TJP2 Reverse	TCTGCCACAAGCCAGGATGTCT
E-cadherin Forward	CGGGAATGCAGTTGAGGATC
E-cadherin Reverse	AGGATGGTGTAAGCGATGGC
β-catenin Forward	AAAGCGGCTGTTAGTCACTGG
β-catenin Reverse	GACTTGGGAGGTATCC ACATCC
CARF Forward	TCAAAGTGACAGATGCTCCA
CARF Reverse	CGTTGAACTGTTTTCCTGCT
Wnt-3α Forward	CAAGATTGGCATCCAGGAGT
Wnt-3α Reverse	TCCCTGGTAGCTTTGTCCAG

c-Myc Forward	AATGAAAAGGCCCCCAAGGTAGTTATCC
c-Myc Reverse	GTCGTTTCCGCAACAAGTCCTCTTC
Cyclin D1 Forward	GAAGATCGTCGCCACCTG
Cyclin D1 Forward	GACCTCCTCCTCGCACTTCT
AXIN Forward	ACTGCCCACACGATAAGGAG
AXIN Forward	CTGGCTATGTCTTTGGACCA
Mortalin Forward	AGCTGGAATGGCCTTAGTCAT
Mortalin Reverse	CAGGAGTTGGTAGTACCCAAATC
Fibronectin Forward	GGAGAATTCAAGTGTGACCCTCA
Fibronectin Reverse	TGCCACTGTTCTCCTACGTGG
Vimentin Forward	CCTTGAACGCAAAGTGGAATC
Vimentin Reverse	GACATGCTGTTCCTGAATCTGAG
hnRNP-K Forward	AGCAGAGCTCGGAATCTTCCTCTT
hnRNP-K Reverse	ATCAGCACTGAAACCAACCATGCC
MMP-2 Forward	TACAGGATCATTGGCTACACACC
MMP-2 Reverse	GGTCACATCGCTCCAGACT
MMP-3 Forward	ATTCCATGGAGCCAGGCTTTC
MMP-3 Reverse	CATTTGGGTCAAACTCCAACTGTG
MMP-7 Forward	GAGTGAGCTACAGTGGGAACA
MMP-7 Reverse	CTATGACGCGGGAGTTTAACAT
MMP-9 Forward	TGTACCGCTATGGTTACACTCG
MMP-9 Reverse	GGCAGGGACAGTTGCTTCT
MMP-13 Forward	AGCAGAGCTCGGAATCTTCCTCTT
MMP-13 Reverse	ATCAGCACTGAAACCAACCATGCC
VEGF Forward	CTACCTCCACCATGCCAAGT
VEGF Reverse	GCAGTAGCTGCGCTGATAGA
CD44 Forward	GGATCCACCCCAACTCCATC
CD44 Reverse	AGGTCCTGCTTTCCTTCGTG
CD24 Forward	CCCACGCAGATTTATTCCAG
CD24 Reverse	GACTTCCAGACGCCATTTG
ABCG2 Forward	TTCTCCATTCATCAGCCTCG
ABCG2 Reverse	TGGTTGGTCGTCAGGAAGA
ALDH1 Forward	CGCAAGACAGGCTTTTCAG
ALDH1 Reverse	TGTATAATAGTCGCCCCCTCTC
OCT-4 Forward	GATGGCGTACTGTGGGCCC
OCT-4 Reverse	TGGGACTCCTCCGGGTTTTG
NANOG Forward	GAGATGCCTCACACGGAGAC
NANOG Reverse	GGTCTGGTTGCTCCACATTG
SOX2 Forward	GCCGAGTGGAAACTTTTGTC
SOX2 Reverse	GTTCATGTGCGCGTAACTGT
CD61 Forward	ATGGGACACAGCCAACAACC
CD61 Reverse	GTGGCACAGGCTGATAATGA
CD9 Forward	ATGATGCTGGTGGGCTTC
CD9 Reverse	GCTCATCCTTGGTTTTCAGC
MRP1 Forward	TCTGGGACTGGAATGTCACG
MRP1 Reverse	CCAGGAATATGCCCCGACTTC
CD133 Forward	GCATTGGCATCTTCTATGGTT
CD133 Reverse	CGCCTTGTCCTTGGTAGTGT
MAPK13 Forward	GAGCGTTACCAGAACCTGTCTC
MAPK13 Reverse	AGTAACCGCAGTTCTCTGTAGGT
PI3K Forward	GTGTTGTGGTCTTTGGCTCCGA

PI3K Reverse	CCATGACTTCGCTTCACAGGCA
PLA2G4F Forward	GAGCCATGTCTTCTCTGTACGG
PLA2G4F Reverse	GTCCCTGTAGAGTGTGGAGATG
CDC42 Forward	TGACAGATTACGACCGCTGAGTT
CDC42 Reverse	GGAGTCTTTGGACAGTGGTGAG
NFATC2 Forward	GATAGTGGGCAACACCAAAGTCC
NFATC2 Reverse	TCTCGCCTTTCCGCAGCTCAAT
PXN Forward	CTGATGGCTTCGCTGTCGGATT
PXN Reverse	GCTTGTTCAGGTCAGACTGCAG
18S Forward	CAGGGTTCGATTCCGTAGAG
18S Reverse	CCTCCAGTGGATCCTCGTTA
